# Dietary patterns related to triglyceride glucose index and risk of type 2 diabetes: a large-scale cohort study

**DOI:** 10.3389/fnut.2024.1510926

**Published:** 2025-01-08

**Authors:** Dong Liu, Ziwei Liu, Yue Wu, Yan Hong, Jinghao Fang, Ying Lu, Guangfei Xu, Peipei Kang, Tong Liu, Li-Hua Chen

**Affiliations:** ^1^Department of Nutrition and Food Hygiene, School of Public Health, Nantong University, Nantong, China; ^2^Institute for Applied Research in Public Health, School of Public Health, Nantong University, Nantong, China; ^3^Department of Anesthesiology, Affiliated Tumor Hospital of Nantong University, Nantong, China; ^4^Institute of Pain Medicine and Special Environmental Medicine, Nantong University, Nantong, China

**Keywords:** type 2 diabetes mellitus, UK Biobank, dietary pattern, triglyceride glucose index, reduced rank regression, LASSO

## Abstract

**Background:**

Triglyceride glucose (TyG) index has been proposed as a credible and simple surrogate indicator for insulin resistance. The primary aim of this study was to novelly examine the associations between dietary patterns reflecting variations in circulating TyG index and the risk of type 2 diabetes mellitus (T2DM).

**Methods:**

This study included 120,988 participants from the UK Biobank, all of whom completed multiple 24-h dietary assessments. Dietary pattern scores were derived using reduced-rank regression (RRR) and Least Absolute Shrinkage and Selection Operator (LASSO) regression, based on the TyG index and approximately 80 food groups. The associations between the TyG index, related dietary pattern scores, and T2DM risk were evaluated using Cox regression models.

**Results:**

During a median follow-up period of 11.2 years, 3,585 participants developed T2DM. A higher TyG index was significantly associated with an increased risk of T2DM. The two dietary patterns derived from RRR and LASSO showed a strong correlation (*ρ* = 0.88, *p* < 0.001) and shared similar characteristics at higher scores, including greater intakes of margarine, meat, fruit juice, and potatoes, alongside lower intakes of green vegetables, oily fish, yogurt, nuts and seeds, and dried fruits. Corresponding blood profiles, including elevated levels of C-reactive protein and HbA1c, along with reduced levels of HDL-C and docosahexaenoic acid, substantiated the dietary pattern assessments. The adjusted hazard ratios (HRs) for T2DM risk were 1.52 (95% CI: 1.33–1.73, *p* trend <0.001) and 1.48 (95% CI: 1.30–1.69, *p* trend <0.001) for dietary patterns derived using RRR and LASSO, respectively, comparing the highest to the lowest quintiles.

**Conclusion:**

The findings suggest that a higher TyG index and specific dietary patterns, characterized by higher intakes of margarine, meat, fruit juice, and potatoes, and lower intakes of green vegetables, oily fish, yogurt, nuts and seeds, and dried fruits, are associated with an increased risk of developing T2DM. These results underscore the potential of dietary modifications targeting these patterns to mitigate T2DM risk.

## Introduction

Type 2 diabetes mellitus (T2DM) is a common metabolic disorder that results in significant morbidity, mortality, and economic burden. Addressing the modifiable risk factors, particularly dietary patterns, is crucial for developing effective prevention strategies (1). Insulin resistance-associated dietary patterns derived from existing dietary index methods have been associated with the risk of T2DM (2, 3).

Dietary patterns have been widely studied for their impact on metabolic health. Recent research, such as that by Gao et al., has examined how high-fat, high-glycemic index, and low-fiber diets are associated with increased T2DM risk by reduced rank regression (RRR) approach (4). RRR is a statistical method used to identify dietary patterns based on biomarkers or nutrient intakes that are known to mediate the relationship between food consumption and the outcome of interest (5). Similarly, Brayner et al. used RRR method to investigate the impacts of different types of dietary fats on obesity and T2DM, highlighting the complex relationships between fat intake and metabolic outcomes (6). The extracted dietary patterns may vary depending on the response variables used by RRR method. Previous studies have used some specific nutrients intake (such as fatty acids and free sugars) as response variables in the RRR model (4, 6). However, nutrient intake assessments are subjective and susceptible to recall bias, which may introduce inaccuracies. Therefore, utilizing objective biomarkers that are closely linked to insulin resistance and reflect overall physiological levels as response variables would be advantageous.

The triglyceride glucose (TyG) index, an indicator derived from the fasting blood glucose and triglyceride levels, has been proposed as a promising and convincing indicator of insulin resistance and suitable for large-scale cohort studies (7, 8). Given the close relationships between dietary factors and blood lipid profiles or glucose levels, it is of high interest to identify dietary patterns associated with the TyG index. Moreover, the TyG index offers several advantages, including simplicity, ease of use, and a strong correlation with hyperinsulinemic-euglycemic clamp measures of insulin sensitivity (9). Despite its potential, the direct influence of dietary components on the TyG index has not been thoroughly explored. This study aims to fill this gap by investigating how different food groups affect the TyG index, thereby providing a more nuanced understanding of dietary patterns on T2DM.

Although the RRR method has been instrumental in deriving dietary patterns (10–15), the Least Absolute Shrinkage and Selection Operator (LASSO) model has recently been proposed for dietary pattern extraction due to its high computational efficiency in improving the prediction of disease-related risk factors (16). However, there exists a notable dearth of studies investigating the relationship between dietary patterns derived from LASSO or in combination with RRR, and the risk of developing T2DM.

In summary, this study focuses on using the TyG index as a response variable and advanced statistical models such as LASSO combined with RRR to identify dietary patterns related to T2DM. The goal is to deepen our understanding of how dietary patterns influence T2DM and provide targeted recommendations for reducing insulin resistance and T2DM risk.

## Materials and methods

### Study subjects

**Figure 1:**
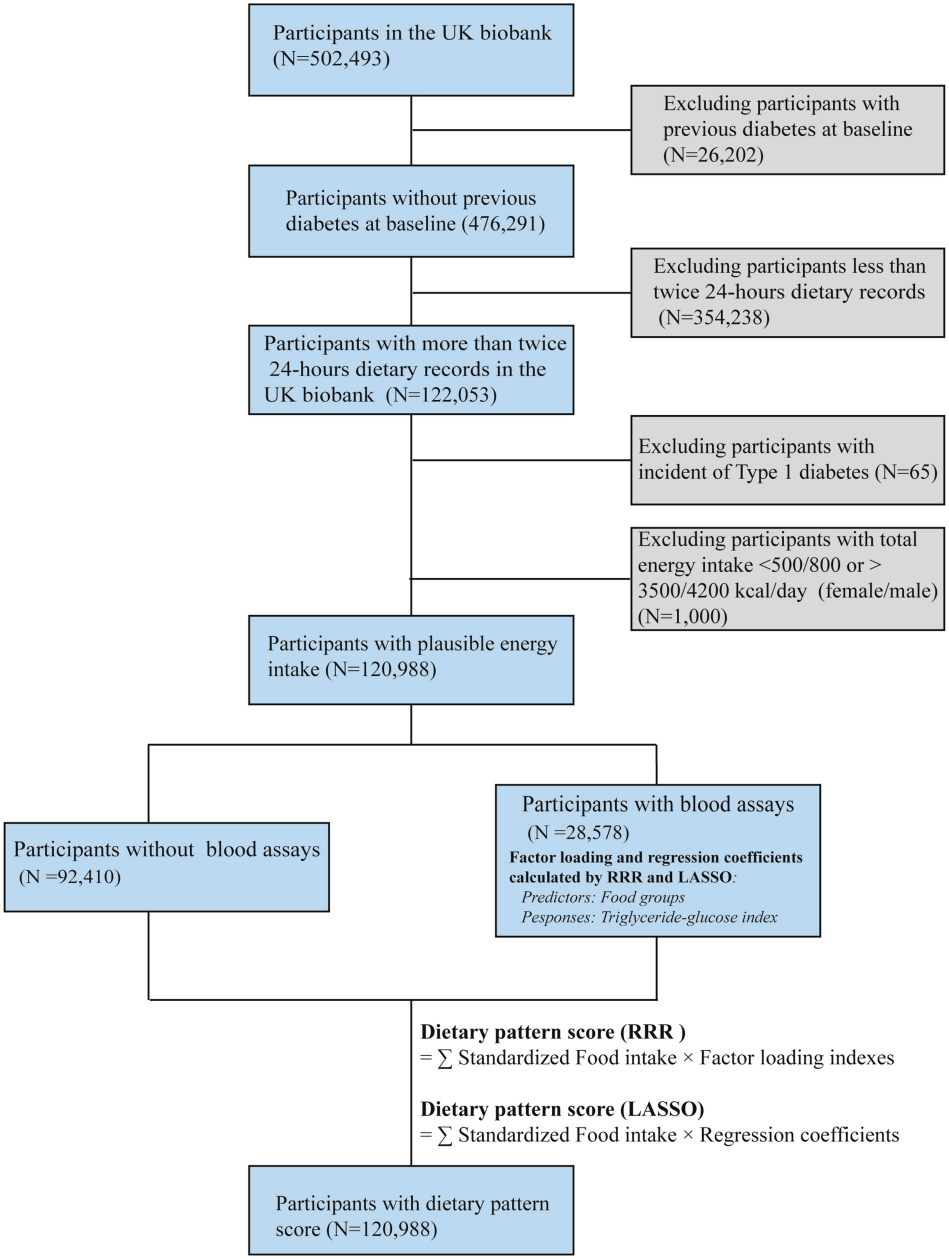
Flowchart of the present study design based on the UK Biobank.

The UK Biobank population-based cohort was recruited between 2006 and 2010 across England, Wales, and Scotland. Extensive phenotypic and genotypic details from 502,493 participants (aged 40–69 years) have been collected (17). Individuals were excluded if they had: (1) a history of diabetes at baseline (*N* = 26,267); (2) only a single or no 24-h dietary record (*N* = 354,238), as multiple dietary records were necessary to capture habitual intake; or (3) implausible energy intake (*N* = 1,000), defined as <500 kcal/day or >3,500 kcal/day for females and <800 kcal/day or >4,200 kcal/day for males, based on established dietary plausibility thresholds (18). After applying these criteria, a total of 120,988 participants were included in the final analysis (Figure 1). A written informed consent was obtained from each participant. All protocols were conducted according to the Declaration of Helsinki.

### Follow-up and outcome definition

The outcome of this study was the incidence of T2DM. Participant follow-up period was defined as from the data of recruitment to the data of first occurrence of outcome death, or 31 September 2020, whichever came first. The date of the first incident diabetes after baseline was ascertained through record linkage with hospital episode statistics in England, Scotland, and Wales and national death registers. The outcome of T2DM was defined using the Tenth Revision of the International Classification of Diseases (ICD-10), with the relevant codes being E11–E14.

### Measurements of biomarkers

Serum biochemical measurements, including glucose, triglycerides (TG), total cholesterol (TC), high-density lipoprotein cholesterol (HDL-C), low-density lipoprotein cholesterol (LDL-C), C-reactive protein (CRP), creatinine, and uric acid, were conducted on a Beckman Coulter AU5800 clinical chemistry analyzer at the central laboratory. HbA1c levels were determined through high-performance liquid chromatography analysis using a Bio-Rad VARIANT II Turbo system. Other biomarker detection used high-throughput NMR-based metabolic biomarker profiling platform developed by Nightingale Health Ltd.

### Covariates

The following covariates were collected at baseline: age, sex, ethnicity, physical activity, Townsend deprivation index, smoking status, drinking status, family history of diabetes, and medication use at baseline. Townsend deprivation index was assigned to negatively represent socioeconomic status (19). Physical activity was derived by Metabolic Equivalent Task (MET-minutes/week) scores (20). Educational attainment was categorized into three groups (high: college or university degree; middle: A/AS levels or equivalent & O levels/GCSEs or equivalent; low: none of the aforementioned). Marital status was defined based on whether the participant was living with a husband, wife, or partner. Body mass index (BMI) was calculated as body weight divided by square of height (kg/m^2^). The calculation of estimated glomerular filtration rate (eGFR, ml/min/1.73 m^2^) was determined by the equation from Chronic Kidney Disease Epidemiology Collaboration (21). Furthermore, cardiovascular disease (CVD) was defined according to the defined stroke and myocardial infarction in the UK Biobank. Hypertension was identified as with ICD-10 records: I10 to 13 and I15, or with recorded SBP/DBP more than 140/90 mm Hg or use of blood pressure-lowering medications. Dyslipidemia was defined as with ICD-10 records: E78, TC > 6.20 mmol/L, TG > 2.30 mmol/L, HDL-C < 1.00 mmol/L, or LDL-C > 4.10 mmol/L or use of cholesterol lowering medication (22).

### Diet assessment

The online self-administered 24-h dietary assessment (Oxford WebQ) for large population studies was used (23). Total energy and nutrient intake data were generated via built-in algorithms and food composition data. Food intakes were categorized into approximately 80 groups based on similarities in their nutritional content or culinary usage. All intakes of food groups and nutrients were adjusted for total energy intake using the residual method (24).

### Calculation of TyG index and identification of dietary patterns

The TyG index was computed utilizing the formula: ln [TG (mg/dL) × glucose (mg/dL)/2] (25). Dietary pattern analyses were conducted using data from participants with blood assays (*N* = 28,578). The “PROC PLS” procedure with the RRR option in SAS was used to calculate factor loadings. The RRR method identified linear combinations of predictor variables, specifically food group intake levels, which explained the variance in TyG index values.

The “glmnet” R package provides access to the LASSO method. In our study, we utilized LASSO on standardized food group intakes to predict TyG index. The dataset was randomly divided into a training set (comprising 75% of the data) and test set. We employed 10-fold cross-validation to determine optimal *λ* for the LASSO construction, aiming to minimize Mean − Squared Error. In the testing set, we employed linear regression model to estimate the adjusted *R*^2^ and correlation coefficient for model comparison (16). The LASSO model assigned six predictors with zero coefficients at optimal “log(*λ*) = −6.14” while yielded a correlation coefficient of 0.65 (*p* < 0.001) and adjusted *R*^2^ of 0.57 for the independent test set.

The correlations of the food groups and derived dietary patterns were quantified by factor loadings for RRR and regression coefficients for LASSO. Subsequently, the derived factor loadings or regression coefficients were projected onto the space of the food groups in the entire cohort (*N* = 120,988) to produce Z-scores, namely, dietary pattern scores (RRR and LASSO).

### Statistical analyses

The normality of continuous data was assessed using the Kolmogorov–Smirnov test. Continuous variables following a normal distribution were presented as means (standard errors), while those not following a normal distribution were presented as medians (interquartile ranges). Categorical variables were expressed as counts (percentages). The Jonckheere-Terpstra test was utilized to assess the *p* trend of continuous variables across quartiles of dietary pattern scores. The Cochran–Mantel–Haenszel test was used for categorical variables. The correlations between dietary pattern scores, TyG index, and other clinical indexes were assessed using Spearman’s correlation coefficients (*ρ*). Cox proportional hazards models with person-year as the time scale were employed to evaluate the risk of T2DM. Hazard ratios (HRs) and corresponding 95% confidence intervals (CIs) were calculated across the quintiles of dietary pattern scores and TyG index (with the first quintile as the reference), and per 1 unit increment of dietary pattern scores. The intakes of food groups were categorized as <1 g or ml per day and quartiles for >1 g or ml per day. *p* for trend was calculated by modeling the categorized number as a continuous variable in the corresponding regression models.

Kaplan–Meier plots with the log-rank test were generated to illustrate cumulative T2DM incidence based on quintiles of dietary pattern scores. Three models were employed to assess the risk associations. Model 1 was adjusted for age, sex, and White race. Model 2 included additional adjustments for physical activity, Townsend deprivation index, educational attainment, marital status, current smoking status, current drinking status, and total energy intake. Model 3 further adjusted for BMI, hyperlipidemia, hypertension, CVD, and family history of diabetes. Restricted cubic spline models with 4 knots were utilized to investigate the potential dose relationships between dietary pattern scores and T2DM. Stratified analyses were conducted to explore potential effect modifications; interaction was evaluated at the multiplicative scale. We conducted several sensitivity analyses to confirm the robustness of our main findings. First, we excluded participants who developed T2DM within the first 3 years of follow-up to mitigate potential reverse causation bias. Second, based on the multivariable-adjusted model, we further adjusted for additional factors including usage of fish oil or vitamin D as supplement (Model A), antihypertensive, antidiabetic, or lipid-modifying agents (yes/no) in the pooled participants (Model B), and biomarkers of eGFR, CRP (Model C), HbA1c, TC, and vitamin D (Model D) as continuous in participants with blood assays. Vitamin D supplement and circulating level were also taken in consideration because of the potential confounding effects (26).

The enhancements in model discrimination and reclassification were additionally assessed with and without each dietary pattern score using integrated discrimination improvement (IDI) and net reclassification improvement (NRI). Discrimination was also assessed using the area under the receiver operating characteristic (ROC) curve (AUC). All statistical tests were two-sided, and significance was determined at a *p*-value of <0.05. Statistical analyses were performed using SAS (version 9.4), and figures were generated using R (version 4.3.1 for Windows).

## Results

### Characterization of dietary patterns derived from RRR and LASSO

**Figure 2:**
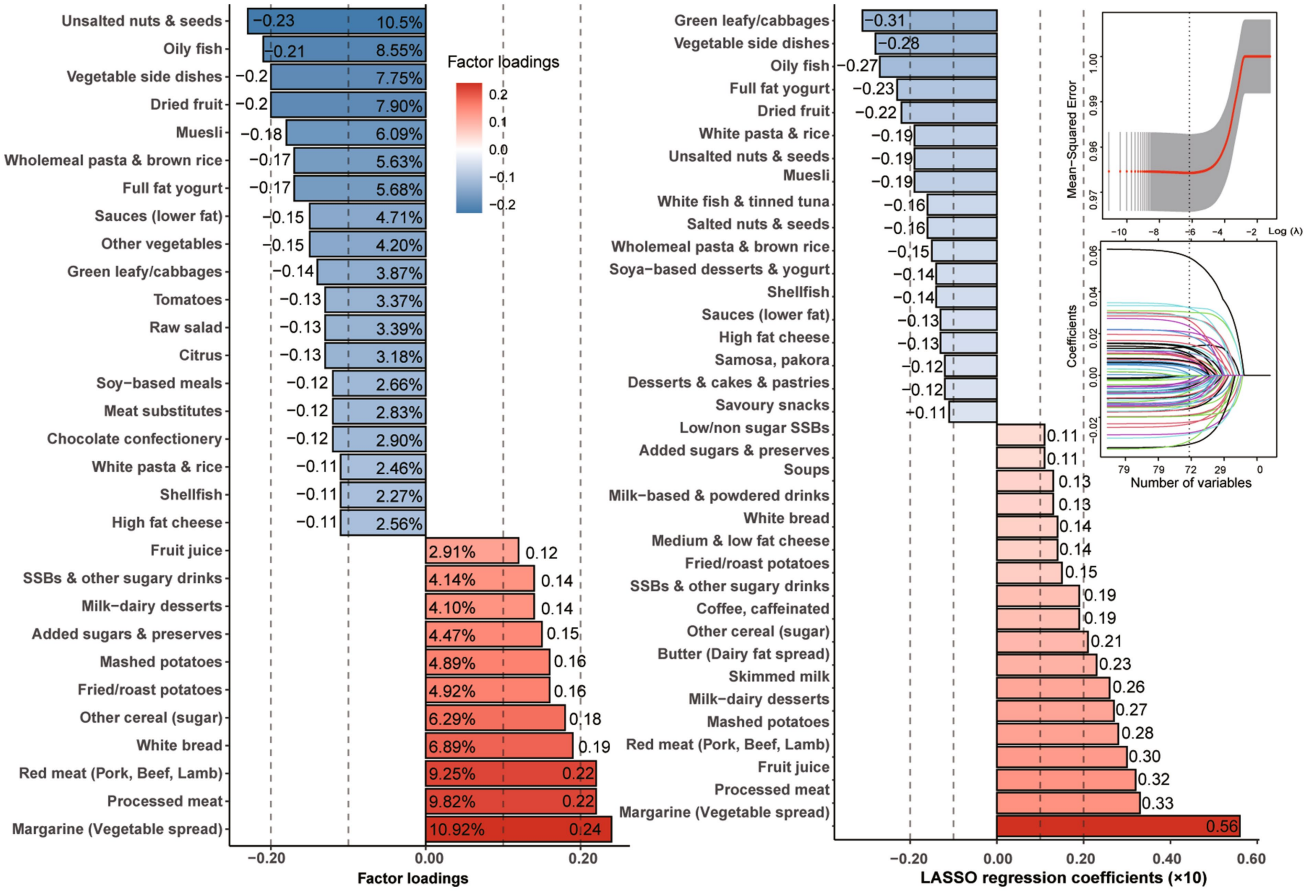
Factor loadings with explainable variation of food groups derived by RRR and regression coefficients with the smallest mean-squared error by LASSO regression.

As depicted in Figure 2 and Supplementary Figure S1, the dietary pattern score derived from RRR was characterized by elevated intakes of margarine (factor loading = 0.24, *ρ* = 0.22), processed meat (0.22, 0.27), and red meat (0.22, 0.28), while exhibiting reduced intakes of unsalted nuts and seeds (−0.23, −0.41) and oily fish (−0.21, −0.32). Similarly, the dietary pattern score-derived prominent food from LASSO was characterized by heighten more intakes, such as margarine (coefficients×10 = 0.56, *ρ* = 0.35), processed meat (0.33, 0.32), fruit juice (0.32, 0.16), red meat (0.30, 0.31), mashed potatoes (0.28, 0.24), milk-dairy desserts (0.27, 0.23), and skimmed milk (0.23, 0.12) while displaying decreased intakes of green leafy/cabbages (−0.31, −0.20), vegetable side dishes (−0.28, −0.29), oily fish (−0.27, −0.29), and full-fat yogurt (−0.23, −0.23).

Furthermore, as shown in Supplementary Figure S2, the extracted dietary factor scores were significantly correlated with the TyG index (*ρ* for RRR = 0.15; *ρ* for LASSO = 0.17) and other clinical indicators, such as CRP (*ρ* for RRR = 0.17; *ρ* for LASSO = 0.16), monounsaturated fatty acids (MUFAs, *ρ* for RRR = 0.14; *ρ* for LASSO = 0.15), and saturated fatty acids (SFAs, *ρ* for RRR = 0.09; *ρ* for LASSO = 0.11; all *p* < 0.001). In contrast, the scores were negatively correlated with docosahexaenoic acid (DHA, *ρ* for RRR = −0.22; *ρ* for LASSO = −0.14; both *p* < 0.001).

### Characteristics of the study population

The characteristics of the pooled population by quintiles of the dietary pattern scores are shown in Table 1. Higher dietary pattern scores were associated with male gender, white race, current smoking, and lower educational level (*p* trend <0.001). Moreover, dietary pattern scores were both positively associated with BMI level, prevalence of CVD, hyperlipidemia and hypertension, as well as total energy intake, fat intake, especially saturated fat intake, while they were negatively associated with nutrient intakes, such as polyunsaturated fat acids (PUFAs) and total carotene (*p* trend <0.001). Specifically, participants with higher dietary pattern score derived from LASSO were more likely to be generally older and have higher total protein intake and less physical activity (*p* trend <0.001).

**Table 1:** Demographic, clinical, and nutritional characteristics by quintiles of dietary pattern scores in the participants with 24-h dietary recording from the UK Biobank (*N* = 120,988).

	Dietary pattern score (RRR)				Dietary pattern score (LASSO)			
	Q1 (*N* = 24,118)	Q3 (*N* = 24,243)	Q5 (*N* = 24,192)	*p* trend^c^	Q1 (*N* = 24,120)	Q3 (*N* = 24,234)	Q5 (*N* = 24,217)	*p* trend^c^
Case (%)	379 (1.57)	657 (2.71)	1,129 (4.67)		381 (1.58)	674 (2.78)	1,129 (4.66)	
Age (years)	57 (50, 62)	57 (50, 62)	56 (49, 62)	<0.001	56 (49, 62)	57 (50, 62)	58 (51, 63)	<0.001
White race (%)	23,099 (95.8)	23,414 (96.6)	23,582 (97.5)	<0.001	22,920 (95.1)	23,492 (97.0)	23,718 (98.0)	<0.001
Male (%)	6,816 (28.3)	10,250 (42.3)	14,951 (61.8)	<0.001	7,446 (30.9)	10,394 (42.9)	13,882 (57.4)	<0.001
BMI (kg/m^2^)	24.5 (22.4, 27.1)	26.0 (23.6, 28.9)	27.1 (24.6, 30.2)	<0.001	24.6 (22.4, 27.2)	26.0 (23.6, 28.8)	30.1 (27.1, 24.6)	<0.001
Current smoking (%)	535 (2.22)	965 (3.98)	2,090 (8.65)	<0.001	656 (2.72)	1,109 (4.58)	1,573 (4.61)	<0.001
Current drinking (%)	22,783 (94.6)	22,942 (94.7)	22,585 (93.5)	<0.001	22,734 (94.4)	22,870 (94.5)	22,736 (94.0)	<0.001
Physical activity (MET-minutes/week)	1,671 (813, 3,159)	1,455 (636, 3,066)	1,704 (817, 3,213)	<0.001	1,899 (993, 3,348)	1,666 (794, 3,146)	1,546 (693, 3,116)	<0.001
Townsend index (%)				<0.001				<0.001
Low	7,250 (30.1)	8,386 (34.6)	7,990 (33.1)		7,179 (29.8)	8,255 (34.1)	8,368 (34.6)	
Moderate	7,696 (32.0)	8,209 (33.9)	8,194 (33.9)		7,563 (31.4)	8,169 (33.8)	8,344 (34.5)	
High	9,145 (38.0)	7,625 (31.5)	7,972 (33.0)		9,349 (38.8)	7,784 (32.2)	7,469 (30.9)	
Educational level (%)^a^				<0.001				<0.001
Low	2,830 (11.7)	4,559 (18.8)	7,131 (29.5)		2,930 (12.2)	4,655 (19.2)	6,842 (28.3)	
Moderate	6,255 (26.0)	8,097 (33.4)	9,413 (38.9)		6,415 (26.6)	8,097 (33.4)	9,144 (37.8)	
High	15,016 (62.3)	11,574 (47.8)	7,632 (31.6)		14,758 (61.2)	11,465 (47.3)	8,216 (34.0)	
Family history of diabetes (%)	4,393 (18.2)	4,760 (19.7)	4,940 (20.4)	<0.001	4,386 (18.2)	4,702 (19.4)	4,985 (20.6)	<0.001
Cardiovasculardisease (%)	308 (1.28)	521 (2.15)	828 (3.42)	<0.001	303 (1.26)	501 (2.07)	855 (3.53)	<0.001
Hyperlipidemia (%)	19,869 (82.4)	20,544 (84.8)	21,236 (87.8)	<0.001	19,915 (82.6)	20,573 (85.0)	21,221 (87.7)	<0.001
Hypertension (%)	3,423 (14.2)	4,567 (18.9)	5,646 (23.4)	<0.001	3,144 (13.0)	4,558 (18.8)	6,035 (24.9)	<0.001
Total energy intake (kcal/day)	2,057 (1,760, 2,399)	2,028 (1,727, 2,375)	2,092 (1,770, 2,459)	<0.001	2,045 (1,741, 2,398)	2,039 (1,735, 2,385)	2,082 (1,771, 2,432)	<0.001
Total protein intake (g/day)^b^	79.1 (70.3, 88.5)	80.9 (72.2, 89.9)	78.8 (70.2, 87.9)	<0.001	78.4 (69.5, 87.8)	80.2 (71.6, 89.3)	80.7 (72.3, 89.7)	<0.001
Total carbohydrate intake (g/day)^b^	253 (229, 278)	246 (221, 270)	244 (220, 268)	<0.001	250 (225, 275)	246 (221, 270)	247 (224, 270)	<0.001
Total fat intake (g/day)^b^	73.6 (64.6, 82.6)	74.9 (66.2, 83.5)	76.7 (68.4, 84.0)	<0.001	74.9 (65.7, 84.0)	75.2 (66.4, 83.9)	75.0 (66.8, 83.2)	0.06
Saturated fat intake (g/day)^b^	26.3 (22.1, 30.8)	28.7 (24.5, 33.2)	30.0 (25.8, 34.4)	<0.001	27.1 (22.7, 31.7)	28.8 (24.5, 33.3)	29.2 (25.1, 33.5)	<0.001
Polyunsaturated fat (g/day)^b^	13.7 (11.0, 16.8)	13.2 (10.5, 16.3)	13.6 (10.8, 16.7)	0.16	13.7 (11.0, 16.9)	13.3 (10.6, 16.4)	13.4 (10.7, 16.4)	<0.001
Total carotene (ug/day)^b^	3,612 (2,401, 5,234)	2,634 (1,658, 3,958)	1,802 (903, 2,974)	<0.001	3,226 (2,056, 4,792)	2,581 (1,548, 3,952)	2,265 (1,262, 3,548)	<0.001
Englyst dietary fiber (g/day)^b^	18.7 (15.6, 22.3)	15.7 (13.0, 18.8)	13.1 (10.6, 15.8)	<0.001	17.6 (14.4, 21.2)	15.6 (12.8, 18.9)	14.2 (11.6, 17.1)	<0.001
Vitamin E intake (mg/day)^b^	10.6 (8.93, 12.6)	8.73 (7.22, 10.5)	7.23 (5.86, 8.84)	<0.001	10.2 (8.40, 12.2)	8.68 (7.07, 10.6)	7.70 (6.26, 9.40)	<0.001
Vitamin D intake (μg/day)^b^	2.85 (1.32, 4.87)	2.16 (1.33, 3.72)	2.00 (1.41, 2.85)	<0.001	2.81 (1.30, 4.89)	2.11 (1.31, 3.61)	2.04 (1.44, 2.92)	<0.001

^aLow: none of the aforementioned; Intermediate: A/AS levels or equivalent, O levels/GCSEs or equivalent; High: college or university degree. bThese nutrient intakes were adjusted for total energy intake using the residual method. cp trend across quintiles of dietary pattern score and characteristics of baseline were examined by Jonckheere-Terpstra test for continuous variables, Cochran-Armitage trend test for binary categorical variables, and Cochran–Mantel–Haenszel test for other categorical variables. BMI, body mass index; MET, metabolic equivalent.^

In addition, clinical characteristics across the quintiles of the dietary factor scores among participants also having blood assays (*N* = 28,578) are presented in Supplementary Table S1. Participants who had higher dietary pattern scores were more likely to have elevated levels of CRP, HbA1c, total fatty acids, MUFAs, SFAs, TG, and TyG index and lower levels of HDL-C, total choline, phosphatidylcholines, omega-3 fatty acids (omega-3s), PUFAs, linoleic acid, and DHA (all *p* trend <0.001). In contrast, there were no significant correlations between LDL-C and these two derived dietary patterns.

### Association of dietary patterns and TyG index with the risk of T2DM

During the median of 11.2-year follow-up, 3,585 incidents of T2DM were recorded. Supplementary Figure S3 demonstrates the cumulative event of T2DM across quintiles of dietary pattern scores. The T2DM risk exhibited a significant increase in higher quintiles for each dietary pattern score (log-rank test: *p* < 0.001). Specifically, the cumulative event of T2DM escalated notably over follow-up time in the fifth quintiles (Q5) of both dietary pattern scores compared to the first quintiles (Q1).

As shown in Table 2, when compared to the Q1 of dietary pattern scores in pooled participants, the adjusted HR for the risk of T2DM in the Q5 was 1.52 (95% CI: 1.33–1.73) for RRR and 1.48 (95% CI: 1.30–1.69) for LASSO. Notably, the corresponding adjusted HR for two independent populations (with and without blood assays) showed similar trends for both RRR and LASSO. Significant results were also observed when using the dietary pattern scores in continuous. The T2DM risk in pooled participants increased significantly per 1 unit increment of dietary pattern scores (HR: 1.17, 95% CI: 1.13–1.22) for RRR and (HR: 1.17, 95% CI: 1.13–1.22) for LASSO.

**Table 2:** Hazard ratio (HR) and 95% confidence interval (CI) for risk of T2DM.

	Cases/N	Model 1	Model 2	Model 3
		Crude model plus demographic characteristics	Model 1 plus sociodemographic and behavior factors	Model 2 plus disease history and health conditions
Participants with 24-h dietary recording (*N* = 120,988)				
Derived by RRR				
Q1	386/24,118	Ref (1.00)	Ref (1.00)	Ref (1.00)
Q3	667/24,243	1.65 (1.45, 1.87)	1.53 (1.35, 1.74)	1.23 (1.07, 1.41)
Q5	1,147/24,192	2.77 (2.46, 3.11)	2.22 (1.96, 2.50)	1.52 (1.33, 1.73)
*p* trend		<0.001	<0.001	<0.001
Dietary pattern score	3,585/120,988	1.46 (1.41, 1.51)	1.33 (1.29, 1.38)	1.17 (1.13, 1.22)
*P*-value		<0.001	<0.001	<0.001
Derived by LASSO				
Q1	391/24,120	Ref (1.00)	Ref (1.00)	Ref (1.00)
Q3	689/24,234	1.67 (1.48, 1.89)	1.55 (1.37, 1.76)	1.24 (1.08, 1.42)
Q5	1,155/24,217	2.62 (2.33, 2.94)	2.22 (1.97, 2.50)	1.48 (1.30, 1.69)
*p* trend		<0.001	<0.001	<0.001
Dietary pattern score	3,585/120,988	1.43 (1.38, 1.48)	1.34 (1.30, 1.39)	1.17 (1.13, 1.22)
*p*-value		<0.001	<0.001	<0.001
Participants with 24-h dietary recording but without blood assays (*N* = 92,410)				
Derived by RRR				
Q1	293/18,383	Ref (1.00)	Ref (1.00)	Ref (1.00)
Q3	528/18,487	1.71 1.48 1.98	1.58 (1.37, 1.83)	1.26 (1.09, 1.45)
Q5	868/18,365	2.75 2.40 3.15	2.18 (1.86, 2.50)	1.47 (1.28, 1.69)
*p* trend		<0.001	<0.001	<0.001
Dietary pattern score	2,754/92,410	1.46 (1.40, 1.52)	1.33 (1.28, 1.39)	1.16 (1.11, 1.21)
*p*-value		<0.001	<0.001	<0.001
Derived by LASSO				
Q1	299/18,481	Ref (1.00)	Ref (1.00)	Ref (1.00)
Q3	531/18,518	1.68 (1.45, 1.93)	1.56 (1.35, 1.80)	1.21 (1.05, 1.40)
Q5	870/18,354	2.58 (2.26, 2.95)	2.17 (1.90, 2.49)	1.43 (1.25, 1.65)
*p* trend		<0.001	<0.001	<0.001
Dietary pattern score	2,754/92,410	1.42 (1.37, 1.48)	1.33 (1.28, 1.39)	1.16 (1.11, 1.21)
*p*-value		<0.001	<0.001	<0.001
Participants with 24-h dietary recording and blood assays (*N* = 28,578)				
Derived by RRR				
Q1	93/5,735	Ref (1.00)	Ref (1.00)	Ref (1.00)
Q3	139/5,756	1.45 (1.11, 1.88)	1.36 (1.04, 1.77)	1.20 (0.89, 1.61)
Q5	279/5,827	2.83 (2.23, 3.60)	2.34 (1.83, 3.00)	1.67 (1.26, 2.22)
*p* trend		<0.001	<0.001	<0.001
Dietary pattern score	831/28,578	1.45 (1.35, 1.57)	1.35 (1.25, 1.45)	1.22 (1.12, 1.33)
*p-*value		<0.001	<0.001	<0.001
Derived by LASSO				
Q1	92/5,639	Ref (1.00)	Ref (1.00)	Ref (1.00)
Q3	158/5,716	1.66 (1.28, 2.14)	1.55 (1.20, 2.01)	1.32 (0.98, 1.76)
Q5	285/5,863	2.73 (2.15, 3.46)	2.38 (1.87, 3.04)	1.56 (1.19, 2.06)
*p* trend		<0.001	<0.001	<0.001
Dietary pattern score	831/28,578	1.44 (1.34, 1.55)	1.37 (1.27, 1.47)	1.20 (1.11, 1.30)
*p-*value		<0.001	<0.001	<0.001
TyG index				
Q1	52/5,702	Ref (1.00)	Ref (1.00)	Ref (1.00)
Q3	129/5,703	2.24 (1.62, 3.10)	2.174 1.516 3.117	1.83 (1.27, 2.63)
Q5	408/5,702	6.82 (5.10, 9.13)	6.36 (4.60, 8.80)	3.93 (2.80, 5.51)
*p* trend		<0.001	<0.001	<0.001
Continuous	831/28,578	2.30 (2.15, 2.46)	2.20 (2.04, 2.37)	2.13 (1.94, 2.33)
*p-*value		<0.001	<0.001	<0.001

Model 1 was adjusted for age (continuous), sex (male or female), and white race (yes or no). Model 2 was further adjusted for physical activity (quartiles, missing data), Townsend deprivation index (quartiles), educational attainment (high: college or university degree; middle: A/AS levels or equivalent & O levels/GCSEs or equivalent; low: none of the aforementioned), marital status: living with husband, wife or partner (yes or no), current smoking (never, previous, current, or missing data), current drinking (never, previous, current, or missing data), and total energy intake (quartiles). Model 3 was further adjusted for BMI (continuous), hyperlipidemia (yes or no), hypertension (yes or no), CVD (yes or no), and family history of diabetes (yes or no). BMI, body mass index; eGFR, estimated glomerular filtration rate. *P* trend was calculated by modeling the quintile numbers as a continuous variable in Cox proportional hazards models, while continuous *p*-value was obtained from the corresponding dietary pattern scores or the TyG index.

**Figure 3:**
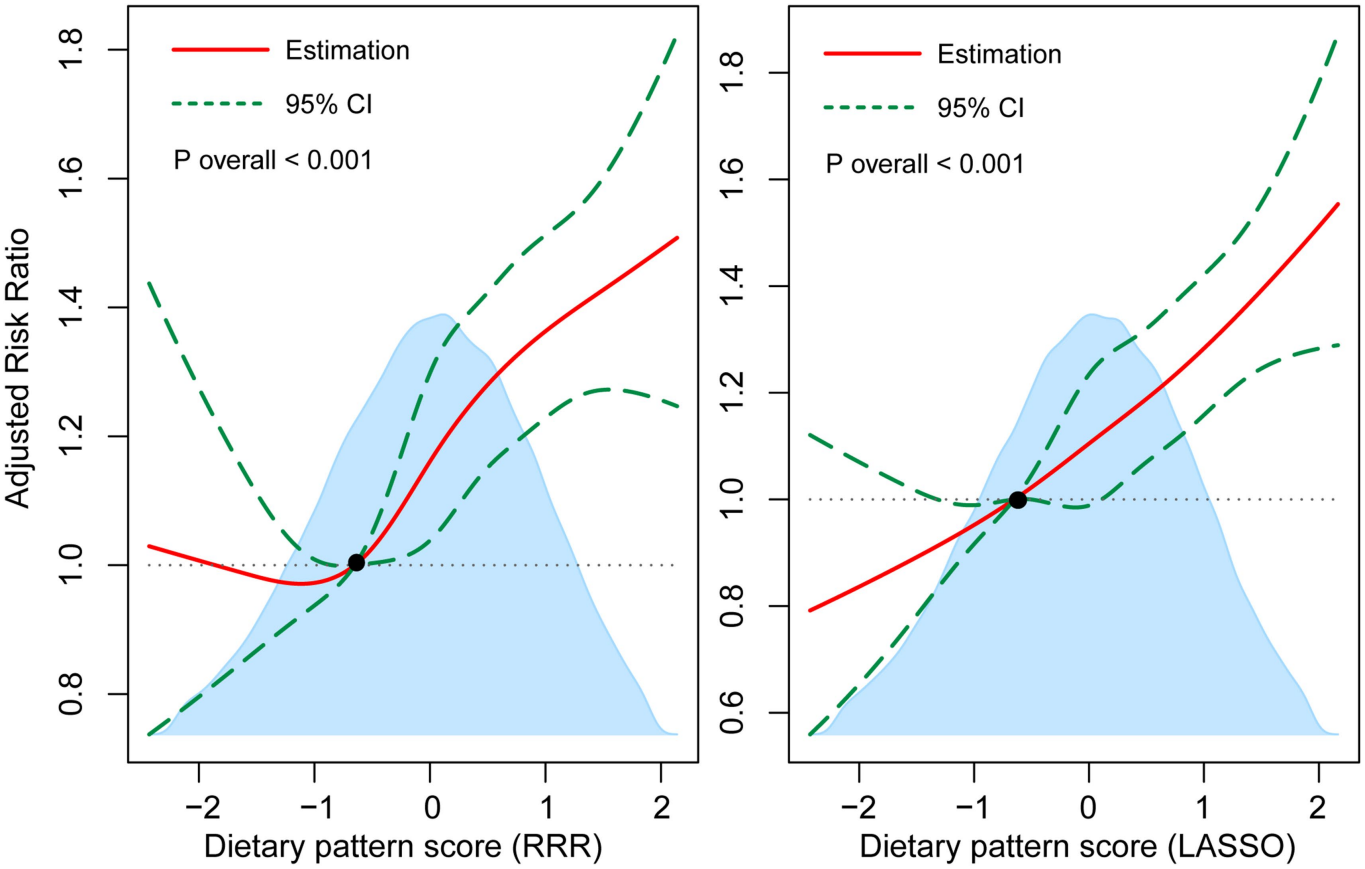
Multivariable-adjusted associations of dietary pattern scores with T2DM incidence by restricted cubic regression. The shaded area illustrates the distributions of dietary pattern scores. The solid lines represent adjusted HRs, while the dotted lines indicate 95% CI. The reference point is the 25th percentile of the reference group from categorical analysis with four knots. Estimates were adjusted for age, sex and white race, physical activity, Townsend deprivation index, educational attainment, living with husband/wife or partner, current smoking, current drinking, total energy intake, BMI, hyperlipidemia, hypertension, CVD, and family history of diabetes.

As shown in Figure 3, multivariable-adjusted restricted cubic spline models both showed positive dose–response relationships between two dietary pattern scores and the risk of T2DM (*p* for linearity <0.001). Moreover, when compared to Q1, the adjusted HR for the risk of T2DM in the Q5 of TyG index was 3.93 (95% CI: 2.80–5.51) as shown in Table 2 for participants with complete blood assays (*N* = 28,578). Consistently, a higher risk of T2DM was observed with an increased continuous level of TyG index (adjusted HR: 2.13, 95% CI: 1.94–2.33).

### Dietary components and their association with T2DM

We further examined individual food groups with factor loadings or regression coefficients (×10) > |0.2| in Supplementary Table S2. Compared to participants with low consumption, those with a full-fat yogurt intake >84.1 mL/day had a significantly reduced risk of T2DM. In contrast, higher intake levels of margarine (>78.1 g/day), red meat (>107 g/day), or processed meat (>46.3 g/day) were associated with an elevated risk of T2DM.

### Model discrimination and reclassification

In reclassification analysis, adding dietary pattern scores derived from RRR or LASSO to the “Model Base 3” (as mentioned in Supplementary Figure S4) significantly improved the category-free NRI by 13.7% (95% CI: 10.0–17.3%) and 13.5% (95% CI: 9.90–17.2%, both *p* < 0.001). Compared to the “Model Base 3,” the discriminative ability of the “Model LASSO” had slight but significant elevation by 0.15% (95% CI: 0.03–0.26%, *p* = 0.016), whereas the difference for the “Model RRR” was at statistical margin (0.11, 95% CI: 0.00–0.23%, *p* = 0.056). In addition, the corresponding IDI for “Model RRR” was 0.11% (95% CI: 0.06–0.15%), and the corresponding index for “Model LASSO” was 0.11% (95% CI: 0.07–0.16%, both *p* < 0.001).

### Stratified and modified analyses

As shown in Supplementary Figure S5, the association between dietary pattern score derived by RRR and T2DM was more pronounced in those participants with younger age, previous smoking, lower physical activity, and without family history of diabetes. In addition, there were significant interactions between dietary pattern score derived by LASSO and T2DM risk modified by race, age, and family history of diabetes.

### Sensitivity analyses

Our primary results remained robust even after excluding participants who developed incident T2DM within the first 3 years (*N* = 596). Compared to the Q1 of the dietary pattern scores derived by RRR and LASSO, the adjusted HRs were 1.56 (95% CI: 1.34–1.80) and 1.57 (95% CI: 1.36–1.81) for Q5, respectively. Moreover, the corresponding risk in such participants increased significantly per 1 unit increment of dietary pattern score for RRR (HR: 1.20, 95% CI: 1.14–1.25) and LASSO (HR: 1.17, 95% CI: 1.12–1.22). As shown in Supplementary Table S3, the robustness of the association between dietary pattern scores and T2DM risk was confirmed in Models A and B. Furthermore, among participants with 24-h dietary records and blood assays, including eGFR, CRP (Model C), TC, HbA1c, and vitamin D (Model D) based on Model 3, the risk associations remained significant, with only minor attenuation.

## Discussion

The dietary patterns associated with TyG index in our study were characterized by higher intakes of margarine, meat, fruit juice, and potatoes and lower intakes of green vegetables, oily fish, yogurt, nuts and seeds, and dried fruits. The TyG index and the dietary patterns derived from it were independently associated with an increased risk of developing T2DM.

Studies have indicated that elevated TyG index was associated with risk of T2DM in Chinese and Brazilian population (27, 28). However, evidence for this relationship in large-scale European populations is still limited, making it essential to study the association between the TyG index and the risk of T2DM. In recent years, there has been a shift toward using subjective metabolite profiles as complementary tools for traditional assessment methods such as 24-h dietary recalls (29). HOMA-IR and HbA1c for insulin resistance have been used as the response variable in RRR (14, 15), but both of whose measurement remains expensive and technically difficult for large-scale cohort (7). Therefore, the TyG index, an easily measured and calculated marker, was used to derive dietary patterns in the present study as it has been proposed as a promising and reliable indicator of insulin resistance (7, 8). We utilized the TyG index as the response variable and identified a dietary pattern characterized by high consumption of margarine, butter, processed meat and red meat, and fruit juice, alongside low intake of oily fish, vegetables, and nuts and seeds. This dietary pattern was found to be significantly associated with T2DM risk. Brayner et al. used fatty acid intake as the response variable and identified two distinct dietary patterns. The first pattern was characterized by higher intake of nuts, seeds, and butter and lower intake of fruit and low-fat yogurt. The second pattern was marked by higher intake of butter and high-fat cheese and lower intake of nuts and seeds (6). However, neither of the dietary patterns was associated with T2DM incidence. The reason for the inconsistency may be that the selected response variable, fatty acid intake, did not fully reflect insulin resistance. Gao et al. further selected energy density, SFA, free sugars, and fiber density intake as response variables. They found that high intakes of chocolate and confectionery, butter, low-fiber bread, sugars, and preserves, as well as low intakes of fruits and vegetables, were associated with higher risk of T2DM (4). Some of features of dietary patterns in our study were generally consistent with previous studies (4, 30, 31). The food groups identified in previous studies are generally consistent with those derived from the TyG index in our study. For example, excessive consumption of red and processed meat (32), added sugars and preserves, mashed potatoes, and low intake of oily fish (33), vegetables, fresh fruit, and nuts (34, 35) have been associated with an increased risk of T2DM. Recent findings from the Atherosclerosis Risk in Communities Study indicated that a dietary pattern minimizing animal-derived foods and emphasizing plant foods may reduce diabetes risk (30). Mozaffarian commented insightfully that when red meat was the only excluded animal-source food (ignoring the consumption of dairy, eggs, fish, poultry, and animal fat), the protective association remained similar. There was a 14% lower diabetes risk across quintiles of the healthy Plant-based Diet Index, compared to the original 15%. This suggests that the lower diabetes risk associated with a healthy plant-based diet is not significantly influenced by the avoidance of these other animal foods (36). This clarification significantly enhances our understanding that not all animal-based foods contribute equally to diabetes risk and underscores the specific impact of red meat. Our current study also supported that higher red and processed meat consumption was associated with higher risk of T2DM, while oily fish consumption was associated with lower risk of T2DM. Therefore, to address hyperglycemia or hyperlipidemia in the context of our findings, dietary interventions should prioritize reducing the intake of margarine, red and processed meat, fruit juice, and potatoes and promoting higher consumption of green vegetables, oily fish, yogurt, nuts, seeds, and dried fruits. Specifically, trans fats in margarine and saturated fats in red and processed meats, identified as key contributors to adverse metabolic profiles, should be replaced with healthier fat sources, such as omega-3-rich fish and unsaturated fats from nuts and seeds. Furthermore, given the significant association between elevated TyG index and T2DM risk, routine monitoring of TyG index in clinical settings could serve as an early marker to guide personalized dietary and lifestyle interventions.

To explore the potential mechanisms, we delved into the metabolic fatty acid profiles, inflammatory marker CRP, and dietary pattern scores. Higher dietary pattern scores, as determined by RRR or LASSO methods, were associated with red and processed meat, added sugars and preserves, and mashed potatoes. Conversely, oily fish, vegetables, fresh fruit, and nuts were associated with lower dietary pattern scores. Our results suggested that both the dietary pattern scores were positively associated with elevated levels of total fatty acids, notably SFAs and MUFAs and CRP. SFAs and MUFAs are predominantly sourced from fatty foods such as red meats, processed meat, butter, and cheese, as well as from margarine (37, 38). SFAs have been widely recognized as a significant factor in the development of T2DM (39). Some studies suggest that high-fat dairy products, particularly butter, could increase the risk due to their saturated fat content (40, 41). Differing from previous studies, both RRR and LASSO analyses in our study found that margarine intake may be the most dangerous food for developing T2DM, as demonstrated in both the dietary pattern analyses and individual food group assessments. During the manufacturing process of hydrogenated vegetable oils, particularly in margarine production, trans fatty acids, specifically trans MUFAs, are generated (42). Artificial trans fatty acid consumption has been shown to decrease HDL-C levels (43), which aligns with our findings of significantly lower HDL-C levels among participants with higher dietary pattern scores. Our results underscored the impact on T2DM risk of dietary composition of fats coupled with commercial prepared processes and other additives.

It is worth noting that our derived dietary pattern by RRR exhibited a moderately negative correlation (*ρ* = −0.13, *p* < 0.001) with the consumption of oily fish, known for its richness in omega-3s (44). Interestingly, the correlation between PUFAs and the derived dietary scores was somewhat attenuated, likely due to the widespread distribution of PUFAs in various food sources (38). However, it is noteworthy that DHA emerged as a notable biomarker indicative of our derived dietary patterns, emphasizing its potential role in modulating metabolic health outcomes. Savolainen et al. have used biomarkers of EPA for fish and linoleic acid for seeds and nuts intakes to identify the potential associations with glucose tolerance status (29). We also noted a stronger correlation between linoleic acid and the dietary pattern score derived from RRR (*ρ* = −0.05, *p* < 0.001) compared to LASSO (*ρ* = −0.02, *p* < 0.001). This discrepancy may be attributed to the stronger influence of nut and seed consumption on the dietary pattern score of RRR. Moreover, increased consumption of nuts pairing dried fruits, providing dietary fibers, and a variety of bioactive compounds could further improve glycemic control (45). Furthermore, the potential effects of these bioactive nutrients were pronounced. For instance, the intake of omega-3s-rich foods may have effects in lowering plasma TC, LDL-C, or CRP (46). Therefore, our results highlight the role of omega-3s, particularly the DHA mainly found in oily fish, in protecting against T2DM.

In our current study, we also found foods controversially associated with T2DM risk, such as skimmed milk. Several studies reported that low-fat dairy products may improve insulin resistance (41) or associate with the decreased T2DM risk (47). However, the evidence regarding these associations is still inconsistent because of the complexes of dairy types and variation in these studies. A recent report suggested that low-fat milk was associated with a higher risk of prediabetes (40). Our findings of dietary pattern derived by LASSO found that individuals who consumed higher skimmed milk may experience an elevation of the T2DM risk. However, when we further examined the relationship between skimmed milk and T2DM, we found no significant association. Slurink et al. further used network estimation found that low-fat milk was usually clustered with energy-dense foods such as bread, meat, and high-fat cheese (40). Therefore, the association between skimmed milk and T2DM may be affected by its clustering with energy-dense foods. Further investigation through long-term, well-designed clinical trials is warranted to explore the causal effects.

The observed associations in our study were stronger among younger adults and individuals without a family history of diabetes, suggesting that dietary interventions tailored to early life stages and specific genetic backgrounds may play a crucial role in preventing T2DM. In addition, as the majority of participants in this study were of White race, the generalizability of our findings to other populations requires further investigation through multicenter cohort studies that include a balanced sample from diverse racial and ethnic groups. Moreover, variations in dietary habits, food availability, and genetic predispositions among different populations may influence the generalizability of these results. In addition, the following limitations should be acknowledged. First, only 23.6% of participants had biomarker test records, which limited the further interpretation of our results. Second, despite efforts to control for confounding variables, residual and unmeasured confounders may still impact the study outcomes due to its observational nature. Meanwhile, several strengths of this study should be highlighted. First, the concurrent use of RRR and LASSO enhances the robustness of the findings, particularly by utilizing the TyG index as a response variable in the causal pathway between food intake and T2DM. Second, the use of repeated dietary assessments helps mitigate recall bias, providing more reliable data. Third, the inclusion of biomarkers allows for objective evaluations of dietary factors, further supporting the validity of the findings.

## Conclusion

In the present study, dietary patterns associated with the TyG index were characterized by higher consumption of margarine, meat, fruit juice, and potatoes, and lower consumption of green vegetables, oily fish, yogurt, nuts, seeds, and dried fruits. These dietary patterns were linked to a higher risk of T2DM, especially among younger adults and individuals without a family history of diabetes.

## Data Availability

Publicly available datasets were analyzed in this study. This data can be found at: this work has been conducted using the UK Biobank Resource under Application Number 62811.
