# Supplementary material for: Dietary patterns related to triglyceride glucose index and risk of type 2 diabetes: a large-scale cohort study
Source: Front Nutr. 2025 Jan 8;11:1510926. doi: 10.3389/fnut.2024.1510926 (PMC11750680; doi:10.3389/fnut.2024.1510926)
Supplement: Supplementary file 1 [file Supplementary_file_1.docx]

## ***Table S1 Clinical characteristics by quintiles of*** ***dietary pattern score in the participants with 24-hour dietary recording and blood assays from the UK biobank (N=28,578).***

|  | Dietary pattern score (RRR) | | | |  | Dietary pattern score (LASSO) | | | |
| --- | --- | --- | --- | --- | --- | --- | --- | --- | --- |
|  | Q1 | Q3 | Q5 | *P* trend |  | Q1 | Q3 | Q5 | *P* trend |
| eGFR (ml/min/1.73 m^2^) | 96.9 (90.2, 103) | 95.5 (88.3, 102) | 95.0 (87.1, 102) | <0.001 |  | 97.3 (90.4, 104) | 95.5 (88.2, 102) | 94.4 (86.4, 101) | <0.001 |
| C-reactive protein (mg/l) | 0.86 (0.44, 1.76) | 1.15 (0.59, 2.32) | 1.47 (0.76, 2.91) | <0.001 |  | 0.85 (0.44, 1.78) | 1.13 (0.59, 2.34) | 1.46 (0.73, 2.86) | <0.001 |
| HbA1c (mmol/mol) | 34.6 (32.3, 36.7) | 34.7 (32.4, 37.1) | 35.0 (32.6, 37.5) | <0.001 |  | 34.4 (32.2, 36.6) | 34.8 (32.4, 37.1) | 35.1 (32.7, 37.5) | <0.001 |
| Total Cholesterol (mmol/l) | 4.69 (4.10, 5.27) | 4.64 (4.05, 5.22) | 4.49 (3.93, 5.09) | <0.001 |  | 4.65 (4.07, 5.24) | 4.62 (4.04, 5.19) | 4.51 (3.94, 5.12) | <0.001 |
| LDL Cholesterol (mmol/l) | 1.71 (1.45, 1.97) | 1.71 (1.46, 2.00) | 1.70 (1.43, 1.99) | 0.38 |  | 1.69 (1.43, 1.96) | 1.71 (1.45, 1.99) | 1.70 (1.43, 1.98) | 0.15 |
| HDL Cholesterol (mmol/l) | 1.41 (1.19, 1.65) | 1.30 (1.10, 1.53) | 1.18 (1.02, 1.39) | <0.001 |  | 1.39 (1.18, 1.64) | 1.30 (1.10, 1.53) | 1.20 (1.03, 1.42) | <0.001 |
| Total Choline (mmol/l) | 2.59 (2.33, 2.86) | 2.54 (2.29, 2.81) | 2.47 (2.22, 2.73) | <0.001 |  | 2.57 (2.31, 2.84) | 2.54 (2.29, 2.80) | 2.49 (2.24, 2.76) | <0.001 |
| Phosphatidylcholines (mmol/l) | 2.13 (1.90, 2.38) | 2.09 (1.86, 2.33) | 2.01 (1.79, 2.25) | <0.001 |  | 2.10 (1.88, 2.35) | 2.08 (1.85, 2.33) | 2.03 (1.81, 2.28) | <0.001 |
| Total Fatty Acids (mmol/l) | 11.3 (10.0, 12.8) | 11.5 (10.2, 13.1) | 11.7 (10.3, 13.4) | <0.001 |  | 11.2 (9.92, 12.7) | 11.5 (10.2, 13.1) | 11.8 (10.3, 13.4) | <0.001 |
| Omega-3 Fatty Acids (mmol/l) | 0.52 (0.39, 0.66) | 0.49 (0.38, 0.64) | 0.47 (0.35, 0.60) | <0.001 |  | 0.52 (0.39, 0.69) | 0.51 (0.38, 0.66) | 0.49 (0.37, 0.63) | <0.001 |
| Omega-6 Fatty Acids (mmol/l) | 4.44 (4.05, 4.89) | 4.44 (4.03, 4.88) | 4.40 (3.97, 4.85) | <0.001 |  | 4.42 (4.01, 4.88) | 4.43 (4.03, 4.87) | 4.42 (4.00, 4.88) | 0.70 |
| Polyunsaturated Fatty Acids (mmol/l) | 5.04 (4.54, 5.57) | 4.98 (4.49, 5.51) | 4.88 (4.39, 5.41) | <0.001 |  | 4.97 (4.48, 5.52) | 4.96 (4.47, 5.49) | 4.92 (4.44, 5.48) | <0.001 |
| Monounsaturated Fatty Acids (mmol/l) | 2.48 (2.11, 2.95) | 2.62 (2.21, 3.15) | 2.79 (2.31, 3.40) | <0.001 |  | 2.45 (2.09, 2.92) | 2.61 (2.21, 3.14) | 2.78 (2.32, 3.38) | <0.001 |
| Saturated Fatty Acids (mmol/l) | 3.76 (3.29, 4.32) | 3.91 (3.41, 4.49) | 3.99 (3.44, 4.66) | <0.001 |  | 3.72 (3.27, 4.28) | 3.90 (3.40, 4.48) | 4.00 (3.46, 4.67) | <0.001 |
| Linoleic Acid (mmol/l) | 3.45 (3.03, 3.93) | 3.40 (3.01, 3.85) | 3.37 (2.95, 3.81) | <0.001 |  | 3.41 (3.01, 3.88) | 3.40 (3.00, 3.83) | 3.38 (2.97, 3.83) | 0.001 |
| Docosahexaenoic Acid (mmol/l) | 0.25 (0.20, 0.32) | 0.23 (0.19, 0.29) | 0.21 (0.17, 0.25) | <0.001 |  | 0.25 (0.20, 0.31) | 0.23 (0.19, 0.29) | 0.21 (0.17, 0.27) | <0.001 |
| Vitamin D (nmol/L) | 48.8 (34.3, 64.1) | 48.8 (35.0, 64.1) | 46.8 (32.8, 61.9) | <0.001 |  | 48.3 (33.5, 64.2) | 48.1 (34.4, 63.6) | 47.8 (33.6, 62.5) | 0.14 |
| Total Triglycerides (mmol/l) | 1.00 (0.75, 1.35) | 1.14 (0.85, 1.54) | 1.29 (0.95, 1.74) | <0.001 |  | 0.98 (0.75, 1.34) | 1.14 (0.84, 1.54) | 1.28 (0.95, 1.72) | <0.001 |
| Glucose (mmol/l) | 3.40 (2.99, 3.82) | 3.40 (2.99, 3.80) | 3.38 (2.97, 3.79) | 0.19 |  | 3.37 (2.95, 3.79) | 3.38 (2.95, 3.81) | 3.41 (3.00, 3.82) | <0.001 |
| TyG index | 7.95 (7.62, 8.29) | 8.00 (7.66, 8.34) | 8.11 (7.76, 8.46) | <0.001 |  | 7.85 (7.52, 8.20) | 7.99 (7.66, 8.34) | 8.12 (7.77, 8.46) | <0.001 |

*P* trend across quartiles of dietary pattern score and characteristics of baseline were examined by Jonckheere-Terpstra test for continuous variables. Abbreviations: HbA1c, Glycated hemoglobin; eGFR, Estimated Glomerular Filtration Rate; LDL, low-density lipoprotein; HDL, high-density lipoprotein; TyG, Triglyceride–glucose index.

## ***Table S2 Hazard ratio (HR) and 95% confidence interval (CI) for risk of T2DM according to quintiles of the*** ***individual food groups.***

| **Food groups** | | **Cases/N** | **HR (95% CI)** |
| --- | --- | --- | --- |
| **Unsalted nuts & seeds (g/day)** | | | |
| Q1 | <1 | 2977/95079 | Ref (1.00) |
| Q2 | (1, 8.18) | 142/6478 | 0.96 (0.79, 1.17) |
| Q3 | (8.19, 13.9) | 171/6477 | 0.99 (0.81, 1.23) |
| Q4 | (14.0, 23.7) | 164/6476 | 0.86 (0.70, 1.06) |
| Q5 | >23.8 | 131/6478 | 0.95 (0.81, 1.11) |
| *P* trend |  |  | 0.40 |
| **Oily fish (g/day)** | | | |
| Q1 | <1 | 2746/86850 | Ref (1.00) |
| Q2 | (1, 37.6) | 192/8535 | 0.97 (0.82, 1.14) |
| Q3 | (37.7, 52.8) | 217/8534 | 0.96 (0.81, 1.14) |
| Q4 | (52.9, 77.6) | 226/8535 | 0.94 (0.81, 1.10) |
| Q5 | >77.7 | 204/8534 | 0.92 (0.80, 1.06) |
| *P* trend |  |  | 0.21 |
| **Dried fruit (g/day)** | | | |
| Q1 | <1 | 2821/88831 | Ref (1.00) |
| Q2 | (1, 10.1) | 208/8040 | 0.91 (0.77, 1.07) |
| Q3 | (10.2, 16.8) | 183/8038 | 0.92 (0.77, 1.10) |
| Q4 | (16.9, 29.1) | 204/8039 | 1.01 (0.86, 1.19) |
| Q5 | >29.2 | 169/8040 | 0.88 (0.76, 1.02) |
| *P* trend |  |  | 0.29 |
| **Vegetable side dishes (g/day)** | | | |
| Q1 | <1 | 1905/58411 | Ref (1.00) |
| Q2 | (1, 38.0) | 476/15644 | 0.94 (0.82, 1.08) |
| Q3 | (38.1, 63.0) | 424/15644 | 1.01 (0.89, 1.14) |
| Q4 | (63.1, 101) | 415/15644 | 0.95 (0.85, 1.08) |
| Q5 | >102 | 365/15645 | 0.93 (0.82, 1.06) |
| *P* trend |  |  | 0.36 |
| **Green leafy/cabbages (g/day)** | | | |
| Q1 | <1 | 1687/50481 | Ref (1.00) |
| Q2 | (1, 37.3) | 469/17626 | 1.07 (0.93, 1.24) |
| Q3 | (37.4, 64.5) | 458/17628 | 0.92 (0.82, 1.04) |
| Q4 | (64.6, 107) | 461/17626 | 0.90 (0.80, 1.02) |
| Q5 | >108 | 510/17627 | 0.94 (0.83, 1.07) |
| *P* trend |  |  | 0.018 |
| **Full fat yogurt (ml/day)** | | | |
| Q1 | <1 | 3005/94143 | Ref (1.00) |
| Q2 | (1, 37.7) | 153/6711 | 1.01 (0.82, 1.23) |
| Q3 | (37.8, 55.8) | 165/6712 | 1.00 (0.81, 1.24) |
| Q4 | (55.9, 84.0) | 141/6710 | 0.91 (0.74, 1.12) |
| Q5 | >84.1 | 121/6712 | 0.83 (0.70, 0.97) |
| *P* trend |  |  | <0.001 |
| **Other cereal (sugar, g/day)** | | | |
| Q1 | <1 | 2586/89764 | Ref (1.00) |
| Q2 | (1, 11.3) | 225/7806 | 0.97 (0.82, 1.15) |
| Q3 | (11.4, 17.8) | 244/7806 | 0.99 (0.83, 1.19) |
| Q4 | (17.8, 26.9) | 260/7806 | 1.10 (0.94, 1.29) |
| Q5 | >27.0 | 270/7806 | 1.09 (0.96, 1.25) |
| *P* trend |  |  | 0.028 |
| **Mashed potatoes (g/day)** | | | |
| Q1 | <1 | 2572/89940 | Ref (1.00) |
| Q2 | (1, 36.8) | 232/7762 | 0.97 (0.81, 1.16) |
| Q3 | (36.9, 49.6) | 260/7762 | 1.02 (0.85, 1.22) |
| Q4 | (49.7, 74.3) | 230/7762 | 1.06 (0.89, 1.25) |
| Q5 | >74.4 | 291/7762 | 1.08 (0.93, 1.25) |
| *P* trend |  |  | 0.08 |
| **Skimmed milk (ml/day)** | | | |
| Q1 | <1 | 2278/81948 | Ref (1.00) |
| Q2 | (1, 23.4) | 334/9760 | 0.85 (0.73, 0.99) |
| Q3 | (23.5, 34.8) | 253/9760 | 0.87 (0.74, 1.02) |
| Q4 | (34.9, 72.1) | 316/9760 | 0.89 (0.77, 1.02) |
| Q5 | >72.2 | 404/9760 | 0.98 (0.86, 1.13) |
| *P* trend |  |  | 0.32 |
| **Fruit juice (ml/day)** | | | |
| Q1 | <1 | 1106/35459 | Ref (1.00) |
| Q2 | (1, 81.9) | 670/21383 | 1.02 (0.91, 1.16) |
| Q3 | (82.0, 166) | 609/21382 | 0.97 (0.86, 1.08) |
| Q4 | (167, 331) | 566/21381 | 1.00 (0.89, 1.13) |
| Q5 | >332 | 634/21383 | 1.08 (0.95, 1.22) |
| *P* trend |  |  | 0.46 |
| **Margarine (g/day)** | | | |
| Q1 | <1 | 1493/58058 | Ref (1.00) |
| Q2 | (1, 31.6) | 455/15733 | 0.89 (0.77, 1.03) |
| Q3 | (31.7, 51.1) | 454/15732 | 1.02 (0.90, 1.16) |
| Q4 | (51.2, 78.0) | 505/15732 | 1.06 (0.94, 1.20) |
| Q5 | >78.1 | 678/15733 | 1.26 (1.12, 1.42) |
| *P* trend |  |  | <0.001 |
| **Butter (g/day)** | | | |
| Q1 | <1 | 1961/64282 | Ref (1.00) |
| Q2 | (1, 25.3) | 366/14177 | 1.04 (0.91, 1.20) |
| Q3 | (25.4, 45.0) | 365/14176 | 0.95 (0.83, 1.09) |
| Q4 | (45.1, 74.6) | 414/14177 | 0.85 (0.76, 0.96) |
| Q5 | >74.7 | 479/14176 | 1.02 (0.90, 1.16) |
| *P* trend |  |  | 0.20 |
| **Milk-dairy desserts (ml/day)** | | | |
| Q1 | <1 | 2141/75619 | Ref (1.00) |
| Q2 | (1, 19.8) | 380/11343 | 0.97 (0.84, 1.12) |
| Q3 | (19.9, 37.0) | 360/11342 | 1.03 (0.87, 1.22) |
| Q4 | (37.1, 69.1) | 332/11342 | 1.06 (0.94, 1.19) |
| Q5 | >69.2 | 372/11342 | 1.06 (0.93, 1.22) |
| *P* trend |  |  | 0.11 |
| **Red meat (g/day)** |  |  |  |
| Q1 | <1 | 1304/49788 | Ref (1.00) |
| Q2 | (1, 40.7) | 544/17800 | 1.14 (0.98, 1.33) |
| Q3 | (40.8, 65.9) | 518/17800 | 1.04 (0.93, 1.17) |
| Q4 | (66.0, 106) | 573/17800 | 1.09 (0.96, 1.23) |
| Q5 | >107 | 646/17800 | 1.26 (1.10, 1.43) |
| *P* trend |  |  | 0.005 |
| **Processed meat (g/day)** | | | |
| Q1 | <1 | 1363/50231 | Ref (1.00) |
| Q2 | (1, 9.5) | 495/17690 | 1.18 (1.01, 1.36) |
| Q3 | (9.5, 21.2) | 517/17689 | 1.08 (0.96, 1.22) |
| Q4 | (21.2, 46.3) | 581/17689 | 1.07 (0.95, 1.21) |
| Q5 | >46.3 | 629/17689 | 1.18 (1.05, 1.34) |
| *P* trend |  |  | 0.09 |

All models were adjusted for age, sex and white race, physical activity, Townsend deprivation index, educational attainment, living with husband/wife or partner, current smoking, current drinking, and total energy intake, BMI, hyperlipidemia, hypertension, CVD and family history of diabetes.

## ***Table S3 Hazard ratio (HR) and 95% confidence interval (CI) for risk of T2DM according to quintiles of the dietary pattern score (RRR&LASSO) of sensitivity analyses.***

|  | Cases/N | Model 3 |  | Model A |  | Model B |
| --- | --- | --- | --- | --- | --- | --- |
|  |  | Model 3, referring to the Table 1 |  | + supplement of fish oil and vitamin D |  | + usage of antihypertensive, antidiabetic, or lipid-modifying agents |
| Pooled: Participants with 24-hour dietary recording (N=120,988) | | | | | | |
| Derived by RRR | | | | | | |
| Q1 | 386/24118 | Ref (1.00) |  | Ref (1.00) |  | Ref (1.00) |
| Q3 | 667/24243 | 1.23 (1.07, 1.41) |  | 1.23 (1.07, 1.41) |  | 1.22 (1.04, 1.40) |
| Q5 | 1147/24192 | 1.52 (1.33, 1.73) |  | 1.51 (1.32, 1.72) |  | 1.51 (1.32, 1.73) |
| *P* trend |  | <0.001 |  | <0.001 |  | <0.001 |
| DP score | 3585/120988 | 1.17 (1.13, 1.22) |  | 1.17 (1.12, 1.22) |  | 1.17 (1.13, 1.22) |
| P value |  | <0.001 |  | <0.001 |  | <0.001 |
| Derived by LASSO | | | | | | |
| Q1 | 391/24120 | Ref (1.00) |  | Ref (1.00) |  | Ref (1.00) |
| Q3 | 689/24234 | 1.24 (1.08, 1.42) |  | 1.24 (1.08, 1.42) |  | 1.24 (1.08, 1.42) |
| Q5 | 1155/24217 | 1.48 (1.30, 1.69) |  | 1.47 (1.29, 1.68) |  | 1.47 (1.30, 1.68) |
| *P* trend |  | <0.001 |  | <0.001 |  | <0.001 |
| DP score | 3585/120988 | 1.17 (1.13, 1.22) |  | 1.17 (1.13, 1.22) |  | 1.17 (1.14, 1.22) |
| *P* value |  | <0.001 |  | <0.001 |  | <0.001 |
|  |  |  |  |  |  |  |
|  | Cases/N | Model 3 |  | Model C |  | Model D |
|  |  | Model 3, referring to the Table 1 |  | + levels of eGFR, C-reactive protein |  | + levels of HbA1c, total cholesterol and vitamin D |
| Participants with 24-hour dietary recording and blood assays (N=28,578) | | | | | | |
| Derived by RRR | | | | | | |
| Q1 | 93/5735 | Ref (1.00) |  | Ref (1.00) |  | Ref (1.00) |
| Q3 | 139/5756 | 1.20 (0.89, 1.61) |  | 1.19 (0.88, 1.61) |  | 1.09 (0.79, 1.51) |
| Q5 | 279/5827 | 1.67 (1.26, 2.22) |  | 1.63 (1.22, 2.18) |  | 1.39 (1.02, 1.89) |
| *P* trend |  | <0.001 |  | <0.001 |  | <0.001 |
| DP score | 831/28578 | 1.22 (1.12, 1.33) |  | 1.20 (1.10, 1.32) |  | 1.14 (1.04, 1.25) |
| *P* value |  | <0.001 |  | <0.001 |  | <0.001 |
| Derived by LASSO | | | | | | |
| Q1 | 92/5639 | Ref (1.00) |  | Ref (1.00) |  | Ref (1.00) |
| Q3 | 158/5716 | 1.32 (0.98, 1.76) |  | 1.32 (0.98, 1.77) |  | 1.28 (0.94, 1.75) |
| Q5 | 285/5863 | 1.56 (1.19, 2.06) |  | 1.53 (1.15, 2.03) |  | 1.29 (0.96, 1.75) |
| *P* trend |  | <0.001 |  | <0.001 |  | 0.017 |
| DP score | 831/28578 | 1.20 (1.11, 1.30) |  | 1.19 (1.10, 1.30) |  | 1.12 (1.02, 1.22) |
| *P* value |  | <0.001 |  | <0.001 |  | <0.001 |

*P* trend was calculated by modeling the quintile numbers as a continuous variable in Cox proportional hazards models, while continuous *P* value was obtained from the corresponding DP scores.

## ***Figure S1 Spearman correlation coefficients (ρ) between dietary pattern score and food group intakes in the UK biobank participants with 24-hour dietary recording (N=120,988).***

**
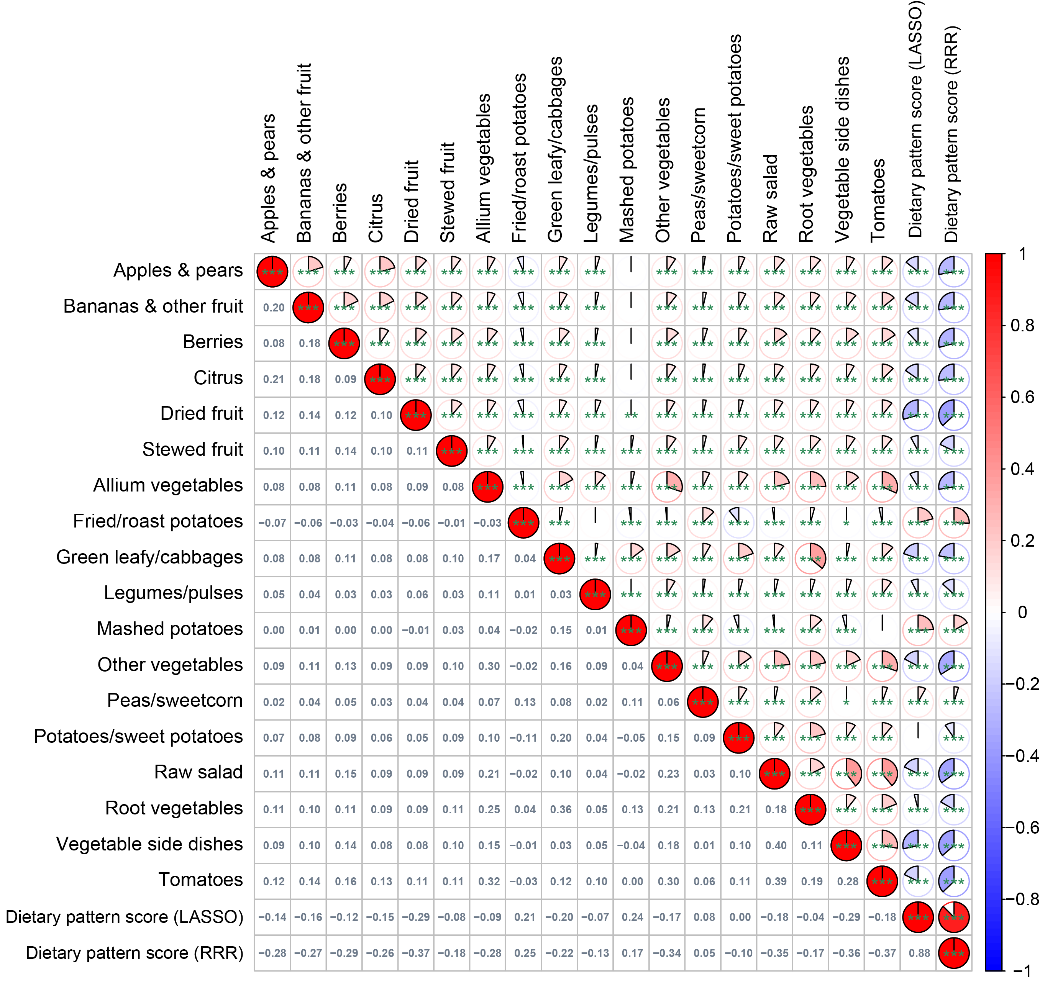

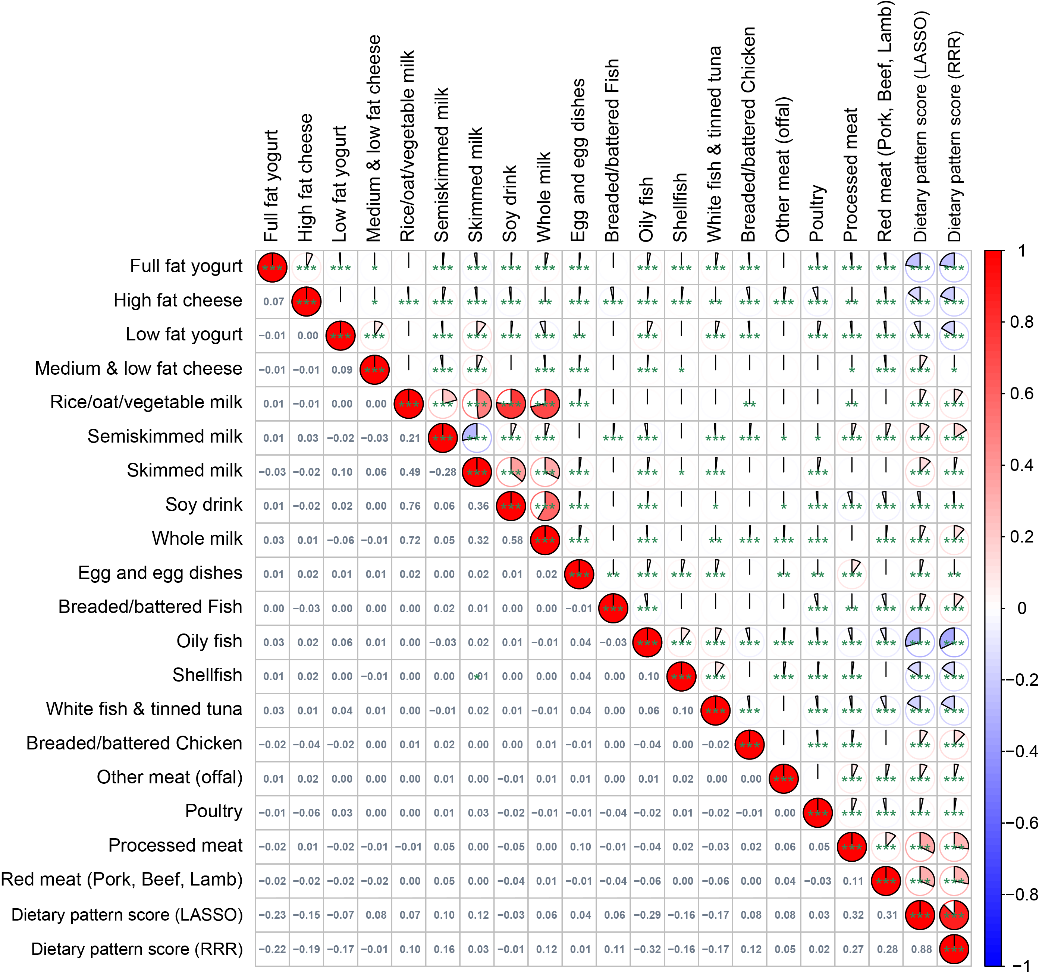
**

**
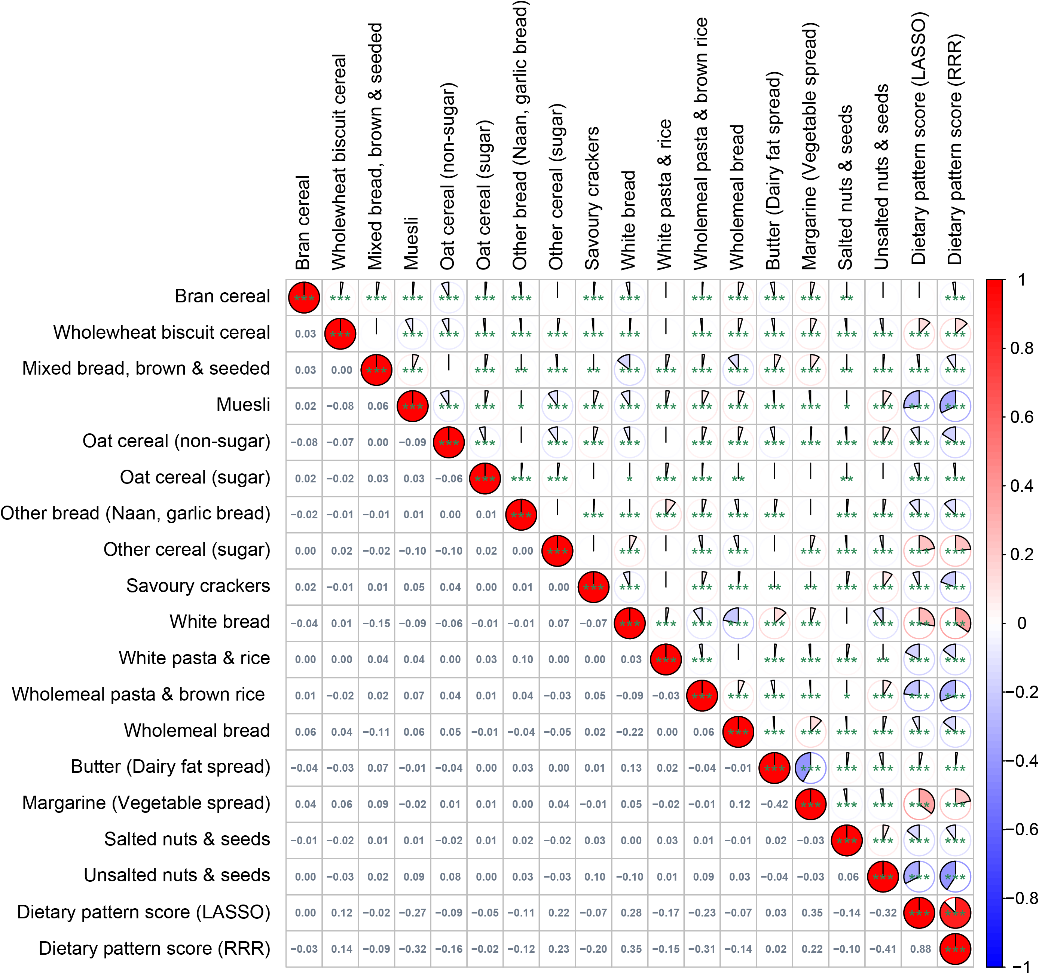

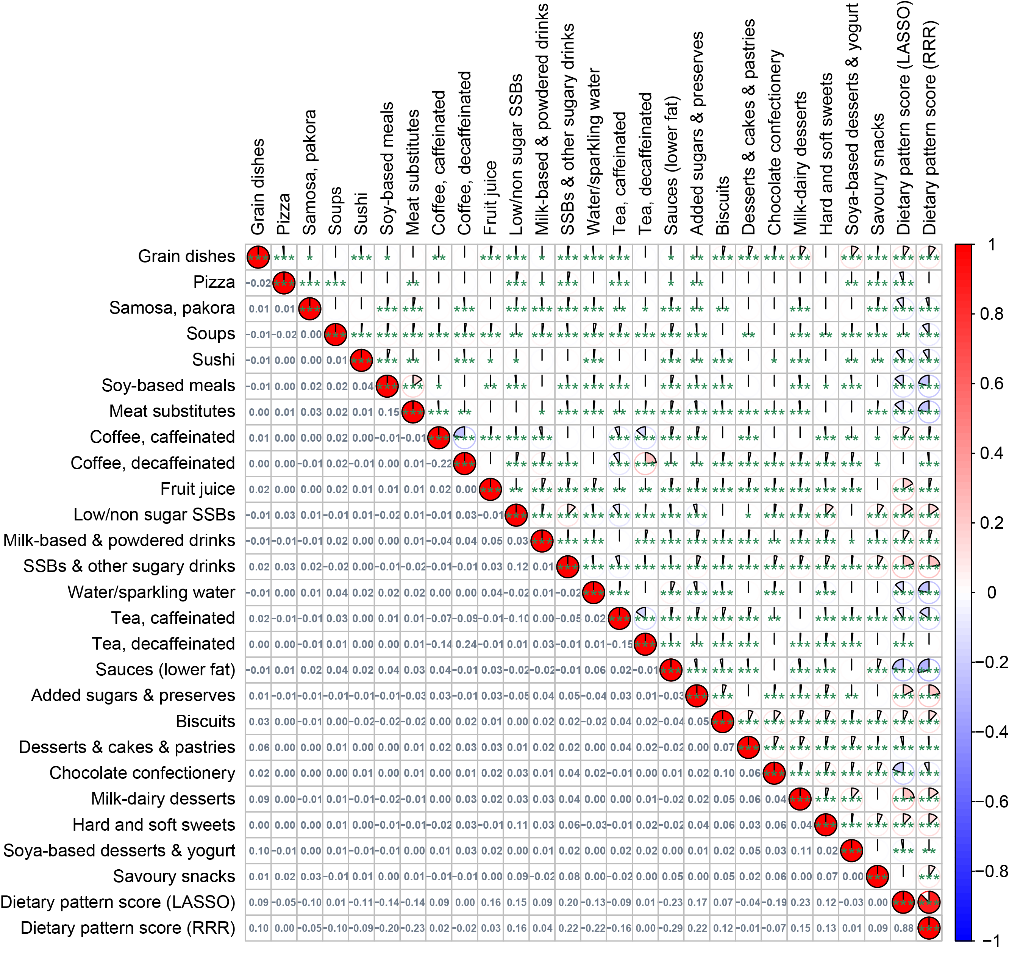
**

## ***Figure S2 Linear regression (β) and spearman correlation coefficients (ρ) between dietary pattern score and clinical indicators in the UK biobank participants with 24-hour dietary recording and blood assays (N=28,559).***


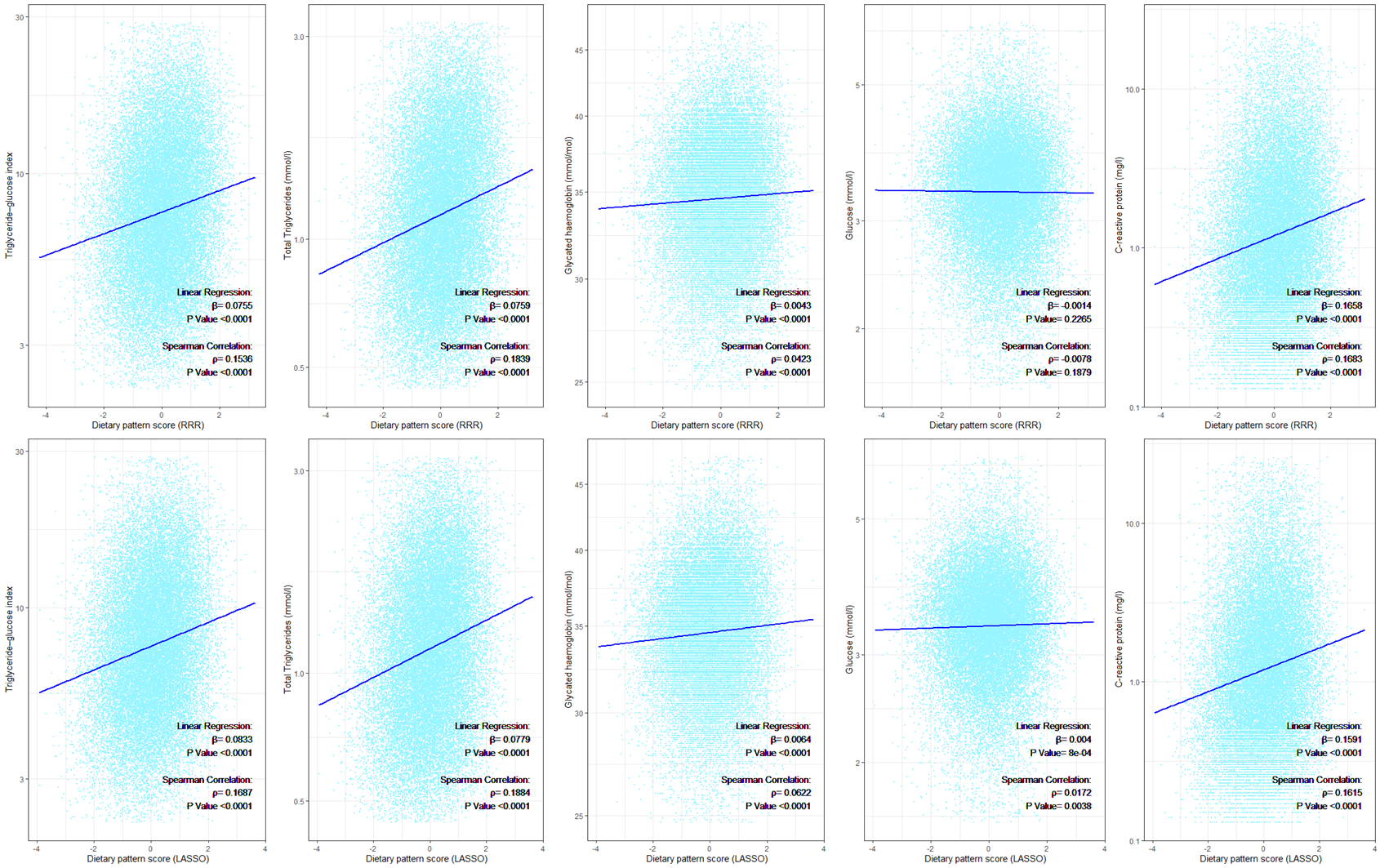


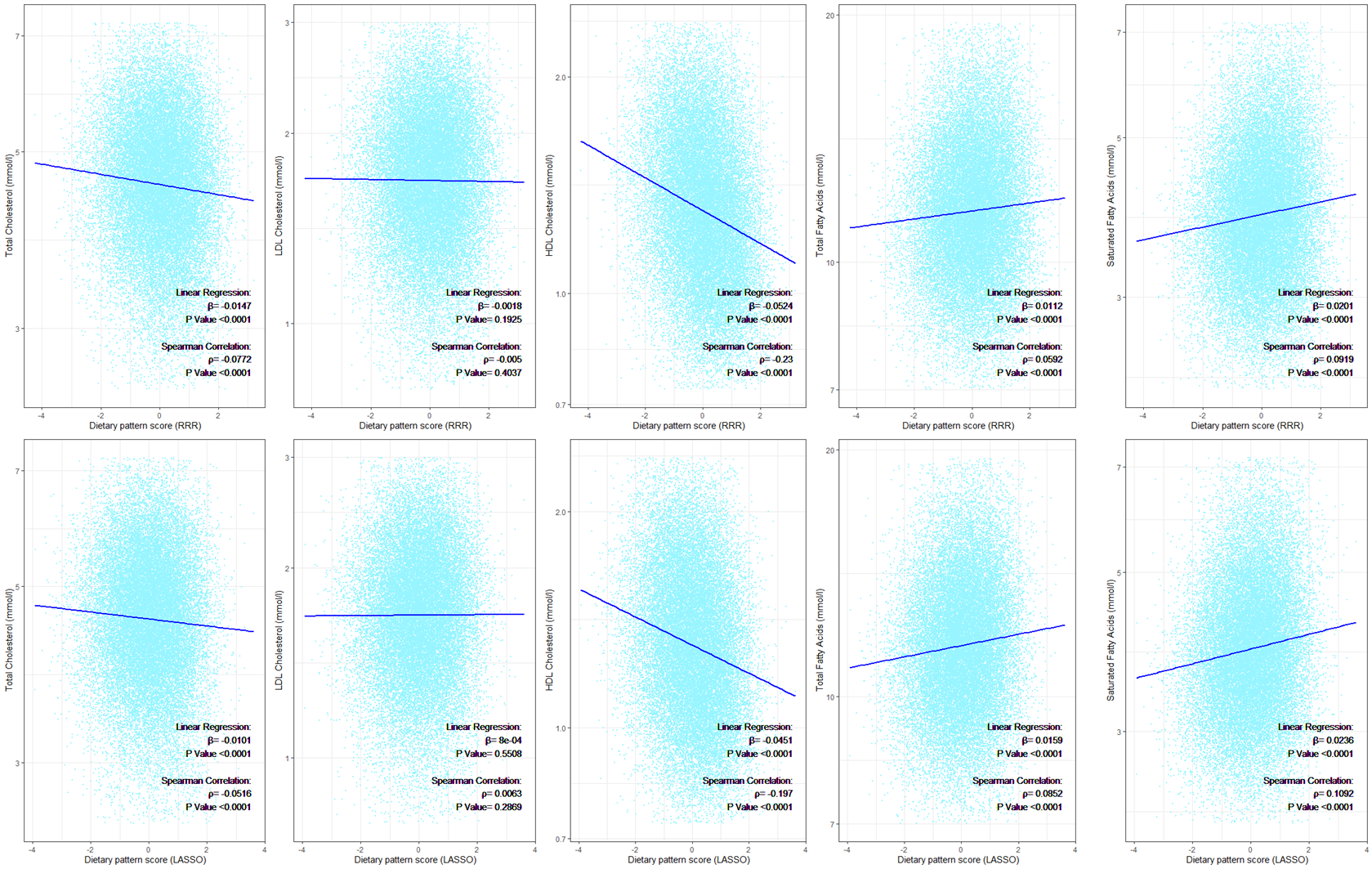


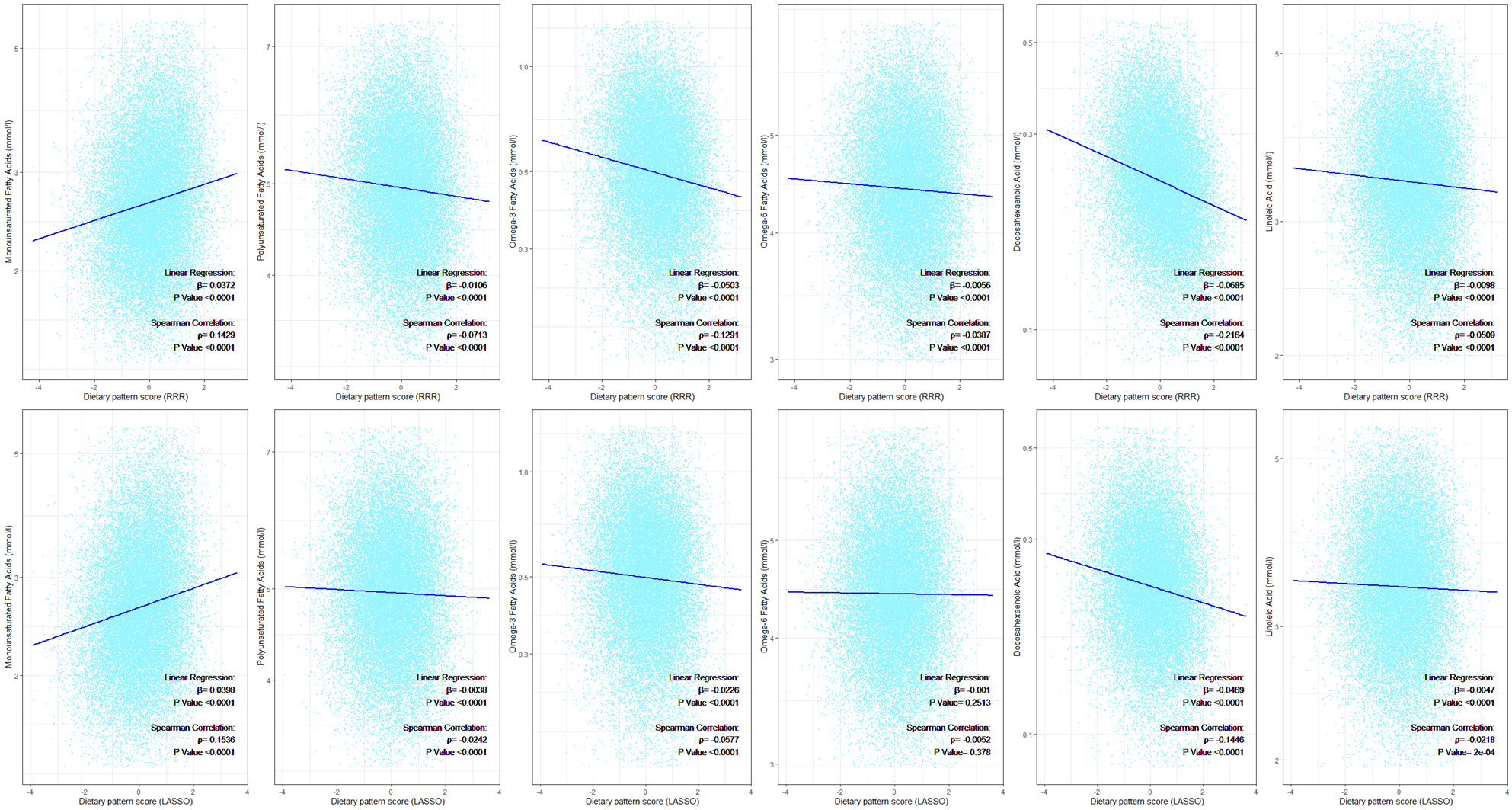


## ***Figure S3 Kaplan-Meier survival curves for cumulative T2DM incidence were analyzed based on quintiles of dietary pattern scores.***


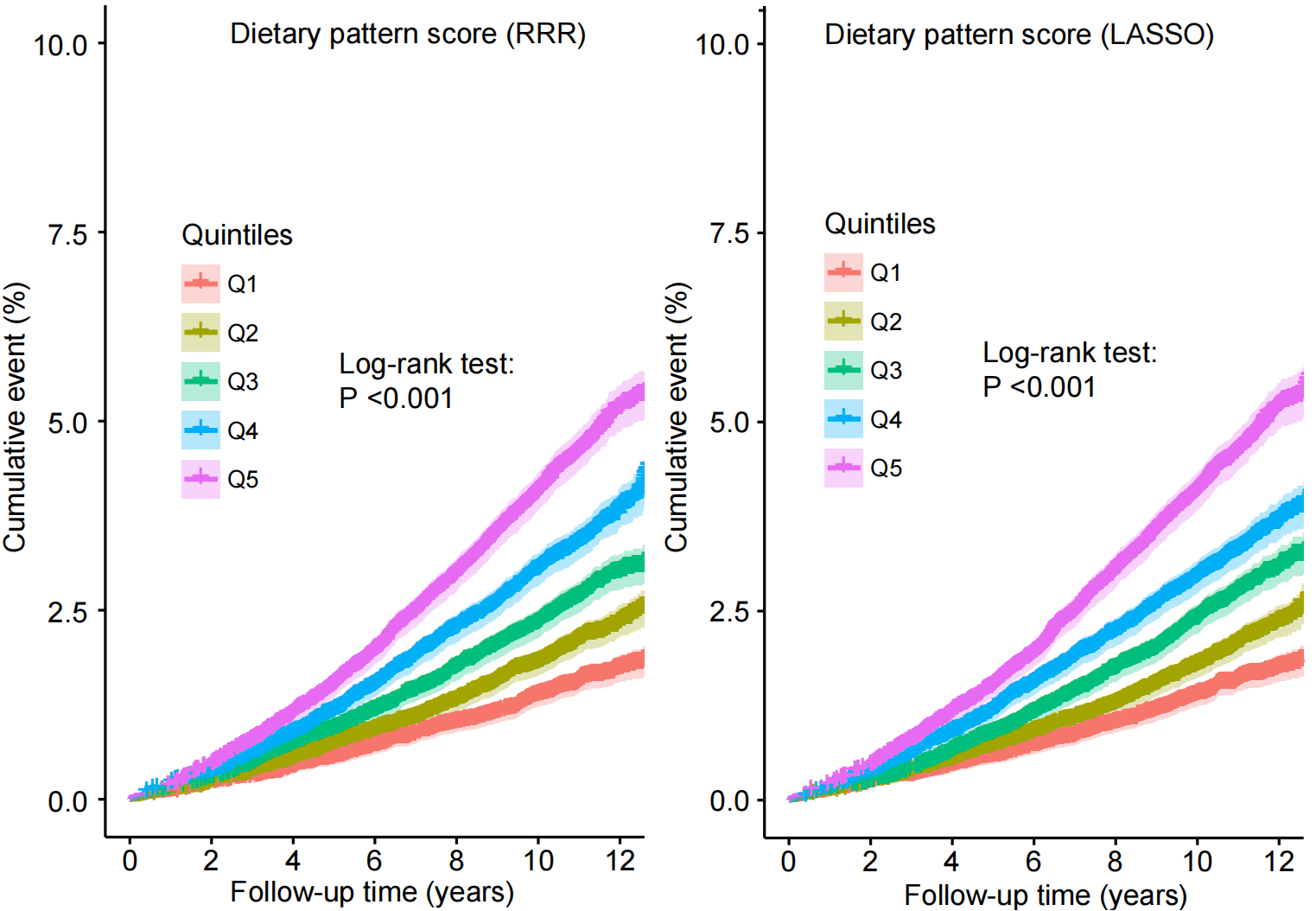


## ***Figure S4 Receiver operating characteristic (ROC) curves were utilized to assess the discrimination performance of models.***

**
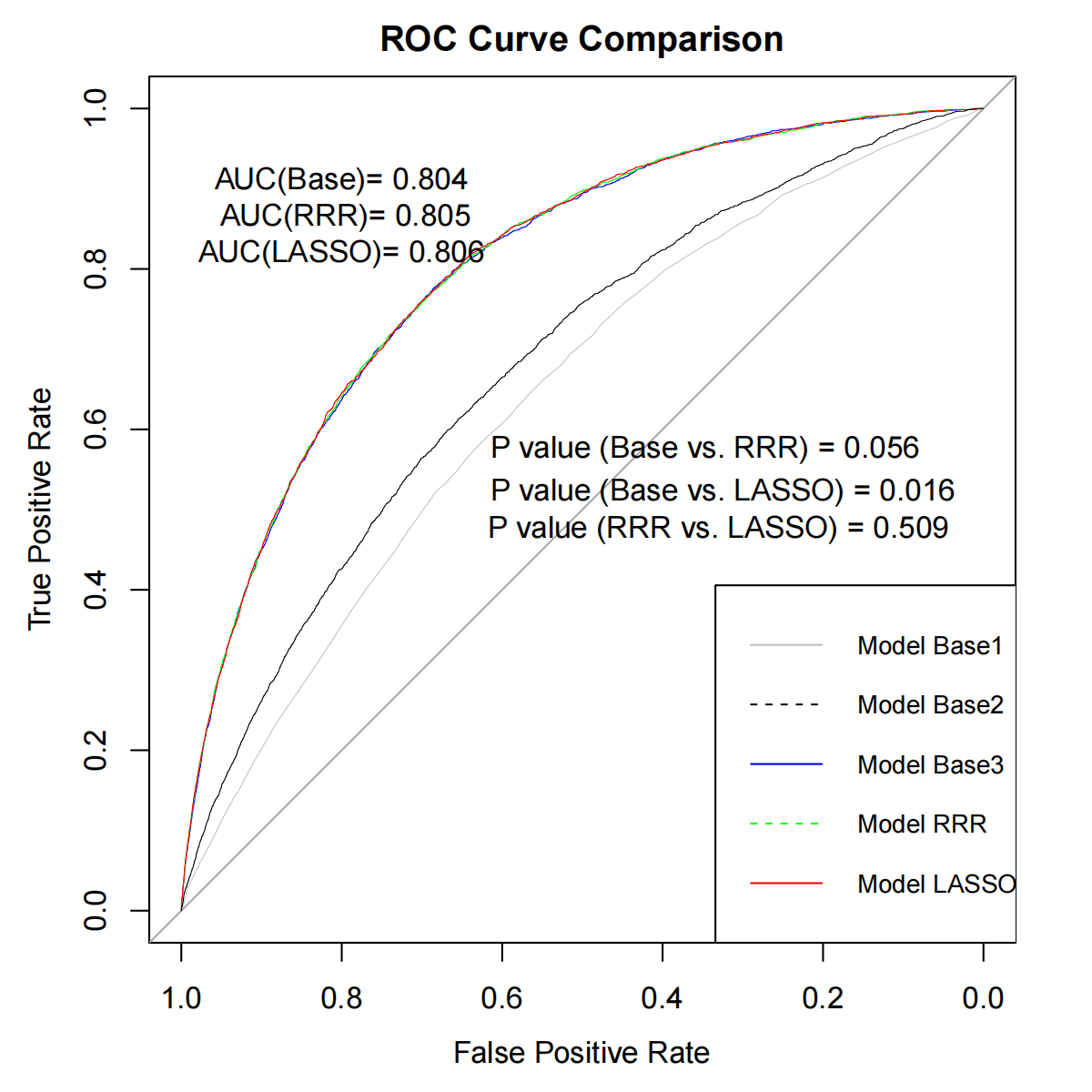
**

Model Base 1: including factors of age, sex and white race; Model Base 2: further including factors of physical activity, Townsend deprivation index, educational attainment, living with husband/wife or partner, current smoking, current drinking, and total energy intake; Model Base 3: further including factors of BMI, hyperlipidemia, hypertension, CVD and family history of diabetes. Model LASSO: including dietary pattern derived by LASSO based on Model Base 3. Model RRR: including dietary pattern derived by RRR based on Model Base 3.

## ***Figure S5 Stratified and modified analyses of the association between the dietary patterns and risk of T2DM.***

***
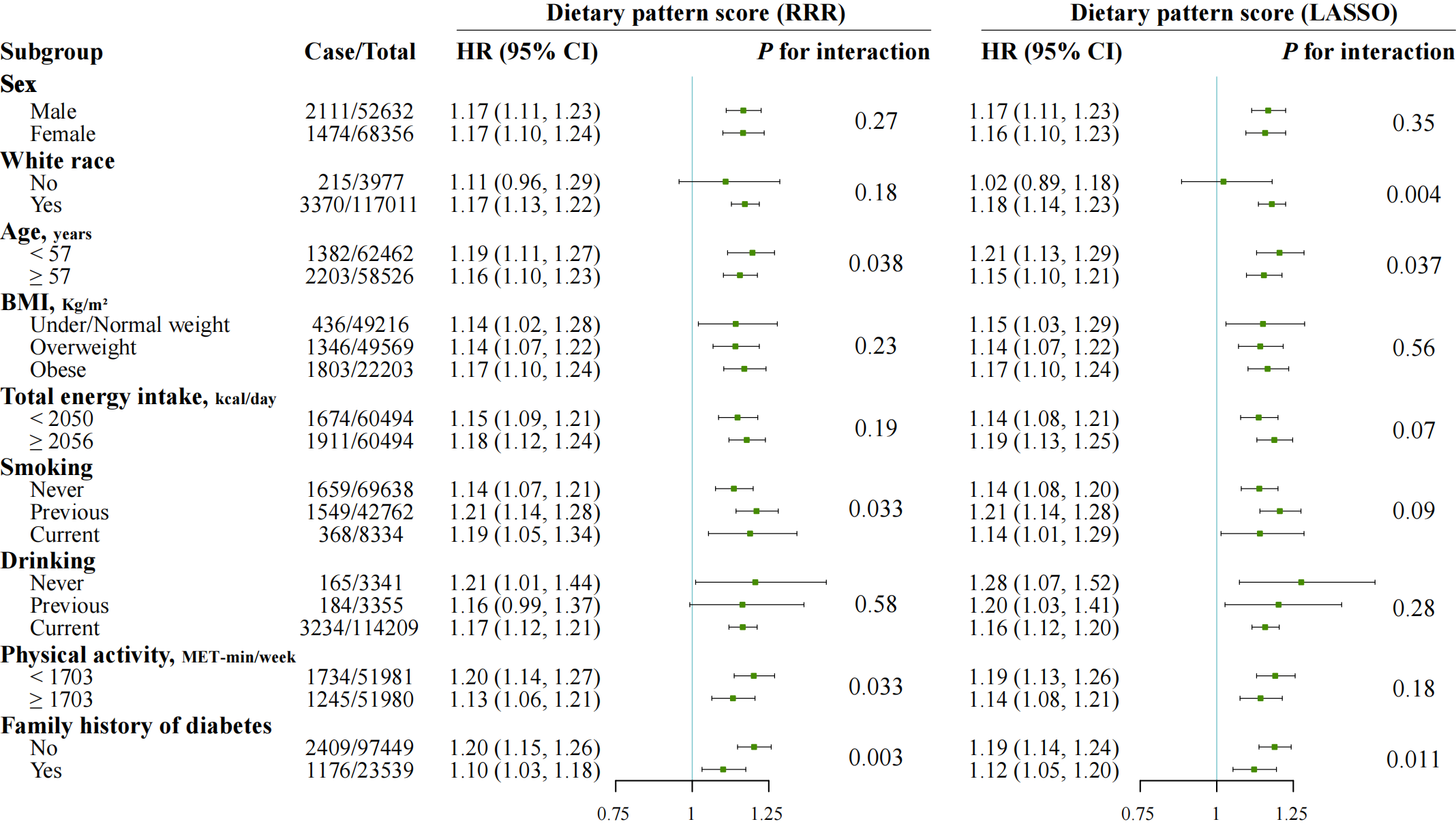
***

Hazards ratios (HRs) and corresponding 95% confidence intervals (CIs) were calculated per 1 unit increment of dietary pattern Z-score values. All models were adjusted for age, sex and white race, physical activity, Townsend deprivation index, educational attainment, living with husband/wife or partner, current smoking, current drinking, and total energy intake, BMI, hyperlipidemia, hypertension, CVD and family history of diabetes. BMI was categorized into three group by <25, ≥30 and <30, or ≥30 Kg/m^2^, other continuous variables were classified by the value of median.
